# Implementation, Challenges, and Outlook of an Intergenerational, Layperson-led, Health Coaching Program (HealthStart): A Pilot Case Study

**DOI:** 10.2196/76592

**Published:** 2025-09-22

**Authors:** Xiaoting Huang, Ka Shing Yow, Audrey Shu Ting Kwan, Jin Ye Yeo, Haikel A Lim, Jie Xin Lim, Meng Han Lim, Lynn Pei Zhen Teo, Nerice Heng Wen Ngiam, Si Qi Lim, Kharuna Jaichandra, Kai Wen Aaron Tang, Angeline Jie-Yin Tey, Lian Leng Low, Kennedy Yao Yi Ng

**Affiliations:** 1 TriGen, Singapore, Singapore; 2 Department of Geriatric Medicine, Singapore General Hospital, Singapore, Singapore; 3 Department of Internal Medicine, National University Health System, Singapore, Singapore; 4 Division of Population Health and Integrated Care, Singapore General Hospital, Outram Road, Singapore, 169608, Singapore, 65 6222 3322; 5 Department of Physiotherapy, Singapore General Hospital, Singapore, Singapore; 6 Department of Psychiatry, National Healthcare Group, Singapore, Singapore; 7 Duke-NUS Medical School, Singapore, Singapore; 8 Primary and Community Care Division, Ministry of Health, Singapore, Singapore; 9 Department of Internal Medicine, Singapore General Hospital, Singapore, Singapore; 10 Department of Respiratory and Critical Care Medicine, Tan Tock Seng Hospital, Singapore, Singapore; 11 Research and Translational Innovation, SingHealth Community Hospitals, Singapore, Singapore; 12 Division of Medical Oncology, National Cancer Centre Singapore, Singapore, Singapore; 13 Department of Global Health and Population, Harvard T.H. Chan School of Public HealthBoston, MA, United States

**Keywords:** healthy aging, health coaching, aging in place, intergenerational, chronic disease management, youth community health volunteers

## Abstract

**Background:**

As rapidly aging populations become a worldwide phenomenon, early detection and prompt management of chronic disease become essential to support healthy aging. Community-based health screenings, a key component of this strategy, often struggle with poor follow-up rates, limiting their long-term impact. Given the untapped potential of youth volunteers and the urgent need for a scalable approach to improve continuity of care post health screenings, we developed HealthStart: a structured, theory-based program that empowers these older adults to take greater ownership of their health and their chronic conditions with the support of youth community health volunteers (CHVs).

**Objective:**

This study aimed to describe the development, implementation, and early outcomes of HealthStart—an intergenerational, layperson-led health coaching program—and summarize operational lessons to guide similar models in Asian communities.

**Methods:**

HealthStart adopted an intergenerational service-learning approach modeled on a self-determination theory–based layperson-led health coaching framework. Each HealthStart team consisted of 1 health care volunteer (HCV) and 4 youth CHVs. All volunteers underwent blended training and were assessed for layperson-led health coaching readiness. Between September 2022 and June 2023 in Singapore, youth CHVs empowered adult participants aged 40 years and older after their health screening to (1) learn about their chronic diseases, (2) learn at least one digital health app, (3) enroll with a primary care provider, and (4) set a lifestyle goal (based on the Specific, Measurable, Achievable, Realistic/Relevant, and Time-bound [SMART] framework for goal setting) and achieve it. We used an implementation-focused case study design using descriptive statistics and volunteer-participant feedback to evaluate feasibility and outcomes.

**Results:**

Of 236 eligible individuals, 192 enrolled. Participants had a mean age of 67 (SD 9.6) years; 52.1% (n=100) of participants were female, with a majority of Chinese ethnicity, having completed primary or secondary school education, residing in self-owned flats, and living in 3-room public housing. Follow-up rate with primary care increased from 42.7% (82/148) preprogram to 84.5% (125/148) postprogram (*χ*^2^_1_=43; *P*<.001). In total, 58 HCVs were recruited, comprising 26 nurses and 6 doctors, with the remainder as allied health professionals. A total of 33 were trained and deployed. The mean age of HCVs was 37 years old, and 24 (72.7%) were female. Furthermore, 149 youth CHVs were recruited, 138 trained, and 102 deployed. The mean age of the youth CHVs who were deployed was 24 years, and 75 (73.5%) were female. Reflections included the importance of volunteer competency and selection criteria, tiering of participant intervention, tapping on community assets, adoption of a social prescription framework, importance of alignment with population health policies, and cultivating intergenerational relationships.

**Conclusions:**

HealthStart demonstrates the feasibility and acceptability of a structured, intergenerational, layperson-led health coaching model embedded in primary care. We identify key lessons learned in the conceptualization and implementation of the program that may inform the design of similar volunteer-enabled initiatives for harnessing laypersons, an often-underused asset, to promote health in the community.

## Introduction

Population aging is accelerating worldwide: by 2030, the number of people above the age of 60 will reach an estimated 1.4 billion [1]. As most years lived with chronic disease accrue after mid-life, timely management of hypertension, diabetes mellitus, and dyslipidemia is pivotal to healthy aging [2]. Effective follow-up after screening enables medication titration, lifestyle counseling, and complication surveillance—cornerstones of chronic-disease control [3].

Singapore, a fast-aging, multi-ethnic, cosmopolitan city-state in Southeast Asia, is no exception. Within the next 5 years, 1 in 4 residents is projected to be 65 years and older, with a corresponding rise in multimorbidity [4]. Community health screenings conducted to identify chronic diseases early report high dropout rates and a lack of follow-up after diagnosis: 1 in 4 people who were screened had not returned for a doctor’s follow-up a year later for their newly diagnosed chronic conditions [5,6]. In response to these challenges, the Singapore government introduced the Healthier SG (HSG) initiative in 2024, which focuses on 5 key features: mobilizing a network of family doctors for preventive care, developing health plans, activating community partners, launching a national enrollment program, and using IT enablers [7]. While welcomed, HSG’s success remains contingent on sustained patient activation, digital literacy, and care navigation gaps that disproportionately affect older adults.

Youth community health volunteers (youth CHVs), as digital natives, are well-placed to support older adults’ use of digital platforms, which play an increasingly important role in health promotion and access to health care [8-10]. In addition, intergenerational service-learning projects in Singapore using youth CHVs such as TriGenerational HomeCare (TriGen) demonstrated reductions in hospital use among older adults [11]. A recent meta-analysis demonstrates that peer coaching by laypersons improved glycemic control and patient activation in chronic disease management [12]. Furthermore, training youth CHVs facilitates the intergenerational transmission of health promotion knowledge and encourages health behaviors during a formative life stage, benefiting both volunteers and their communities [13].

In response to poor postscreening follow-up and the benefits of using lay CHVs, we developed the HealthStart, an intergenerational, youth-led, layperson health-coaching program that provides longitudinal follow-up support to participants after screening. HealthStart mobilizes youth CHVs to help adults aged 40 years and older in achieving the following goals: (1) increase follow-up with a primary care physician, (2) increase ownership of one’s health, (3) increase knowledge of their chronic conditions, (4) increase digital health literacy, and (5) set specific health goals related to their condition and follow through on them. By fostering health literacy, digital engagement, and continuity of care, HealthStart aims to enhance chronic disease follow-up while building intergenerational relationships and bolstering community capacity.

HealthStart mobilizes community assets, including grassroots organizations and trained youth CHV, as part of a scalable, layperson-led model to reinforce continuity of care. In this way, HealthStart functions as a community-based program that extends Healthier SG’s population health objectives into the neighborhoods, particularly among hard-to-reach older adults identified through screening programs.

In this paper, we present the HealthStart program’s conceptual foundations, describe its implementation, and reflect on key operational and training lessons. We also share the experiences of youth CHVs and health care volunteers (HCVs) involved, with the aim of informing similar interventions targeting community-dwelling older adults.

## Methods

### Overview

This study used an implementation-focused case study design to examine the feasibility, operational processes, and outcomes of the HealthStart program over 2 cycles between September 2022 and June 2023. Quantitative and qualitative data were collected to describe participant outcomes and volunteer deployment. The study followed the StaRI (Standards for Reporting Implementation Studies) checklist and the TIDieR (Template for Intervention Description and Replication) extension for complex interventions [14,15].

HealthStart is an initiative by TriGen Ltd [16], a nonprofit organization in Singapore, in collaboration with the Singapore General Hospital Division of Population Health and Integrated Care. The Singapore General Hospital Division of Population Health and Integrated Care is responsible for the population health needs of the approximately 300,000 residents living in the Southeast region [17] and conducts health screening in the community as part of the government’s “Screen for Life” initiative [18]. Individuals aged 40 years and older are invited to attend health screenings conducted in community spaces such as community centers or active aging centers. After undergoing the health screenings, those with ≥1 newly detected or suboptimally controlled chronic condition were invited to participate in the HealthStart program.

### Conceptual Framework

HealthStart is a layperson-led health coaching program grounded in self-determination theory (SDT), incorporating the SMART (Specific, Measurable, Achievable, Realistic/Relevant, and Time-bound) framework for goal setting, and delivered through an intergenerational service-learning model. SDT postulates that there are 3 innate psychological needs—competence, autonomy, and relatedness—which, when satisfied, yield enhanced self-motivation and, when thwarted, lead to diminished motivation and well-being [19]. Goal setting via the SMART framework has been theorized to facilitate behavior change by assisting individuals in defining clear and actionable goals [20]. HealthStart aims to increase the engagement and follow-up rates of participants attending a chronic disease community health screening by developing, training, and empowering CHVs to serve as health coaches. Over a 3-month period, CHVs support participants in their journey to improve their health. The program is designed to promote participants’ autonomy by allowing them to identify goals that are important to them and develop personalized action plans. It enhances competency through SMART goal setting and motivational interviewing, which is taught to all CHVs [21]. Finally, the program’s longitudinal and relational nature emphasizes building connections and fulfilling participants’ need for relatedness.

Volunteer training in HealthStart is anchored upon an intergenerational and service-learning approach. Intergenerational theory focuses equal attention on the potential development of all members of each intergenerational dyad to learn as a function of social interaction [22]. Contact theory, a widely cited theory guiding intergenerational program development and study, describes 4 main tenets that increase the likelihood that people will experience positive contact: support of governing bodies, equal group status, intergroup cooperation, and common goals. Service learning encompasses the teaching approach that connects theory and practice by giving learners the opportunity both to participate in a service that meets community needs and to reflect on the experience to gain a deeper understanding and an enhanced sense of civic engagement [23]. Youth CHVs have the opportunity to acquire health care and caregiving skills that could improve their own health and promote transfer of this health-promoting knowledge to their own communities. In the design of the HealthStart program, we built upon the aforementioned frameworks and lessons learned from a previous intergenerational program that was demonstrated to increase health literacy and reduce ageist attitudes [11]. HealthStart activates 2 additional contact theory principles. Equal status is fostered by training all volunteers together and holding them jointly accountable for participant outcomes, while cooperation toward shared goals is achieved when youth CHVs and participants co-create individualized action plans. Throughout the 3-month journey, HCVs serve as clinical mentors, providing guidance while enabling youth CHVs to lead participant engagement.

**Figure 1.**
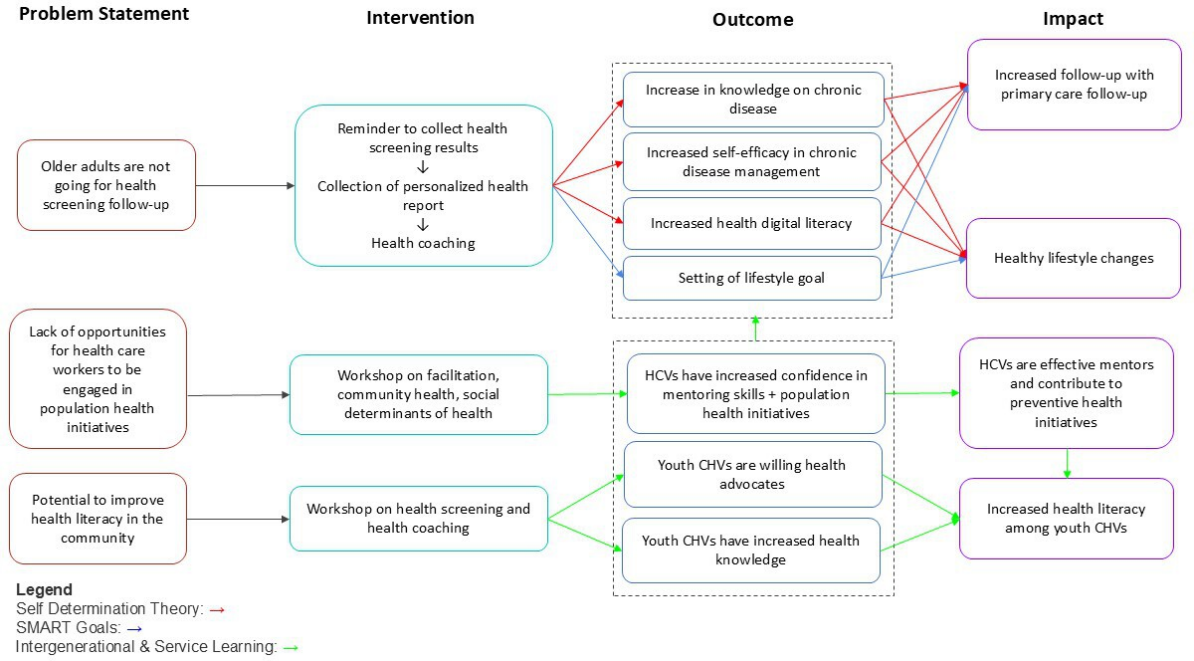
Theory of change as applied to the HealthStart program, with the 3 components—self-determination theory, SMART framework, and intergenerational and service learning—demarcated by different colored arrows across the 4 stages. HCV: health care volunteer; CHV: community health volunteer; SMART: Specific, Measurable, Achievable, Realistic/Relevant, and Time-bound.

SDT served as the overarching framework, providing the central mechanism for fostering self-directed, value-driven motivation. Within this framework, contact theory was used to promote equal-status interactions, service-learning pedagogy to encourage civic engagement, and the transtheoretical model to guide stage-matched health coaching. These components were deliberately integrated into a unified theory of change, with each reinforcing the others, to inform both volunteer training and participant-facing activities and ensure a synergistic approach to sustaining behavior change (see Figure 1).

### Program Description

**Figure 2.**
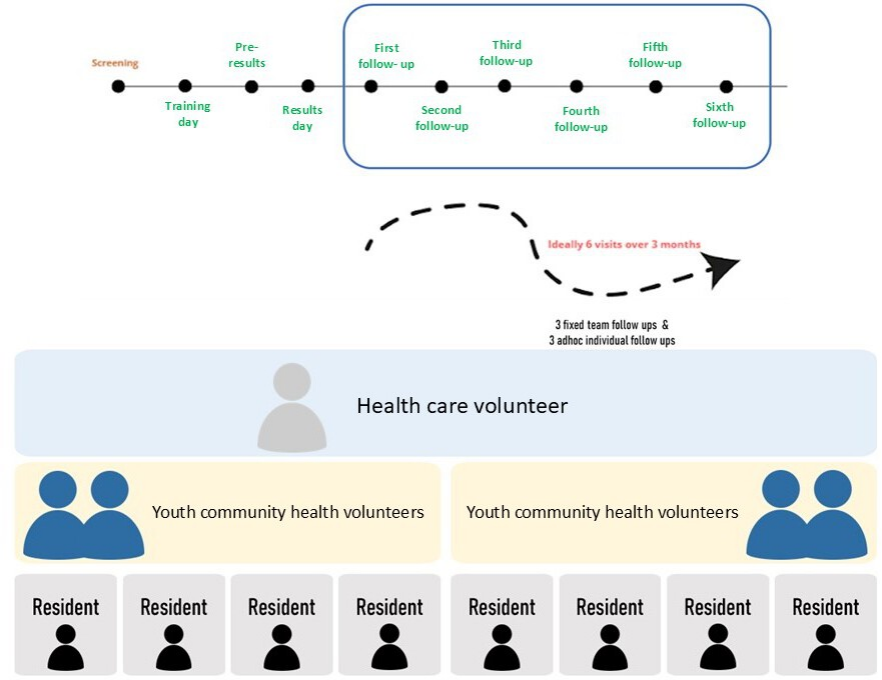
Structure of the HealthStart program for the youth community health volunteers and residents across 3 months with recommended visit types and fixed team follow-ups for team bonding.

Each HealthStart group consists of 1 HCV and 4 youth CHVs who commit to a period of 3 months. Each youth CHV is required to attend the HealthStart training day and is assigned 2 participants subsequently. They are expected to follow up with participants through either physical or internet-based visits (see Figure 2). With each visit, youth CHVs are required to monitor participants’ progress toward their health goals. HCVs oversee the teams, provide avenues for content clarification, and professional support as necessary. This multimodal, layperson-led health coaching aims to facilitate participants’ need for competence (via greater knowledge about their condition and health-efficacy and increased eHealth knowledge), relatedness (via continual interaction with volunteers and health care professionals), and autonomy (via SMART goal setting).

Participants’ baseline health literacy and digital skills were assessed during the initial layperson-led health coaching session using the HealthStart First Visit Form (Multimedia Appendix 1). This structured form captured participants’ understanding of their chronic conditions, experience with digital devices, familiarity with health apps (eg, Healthy365 by the Health Promotion Board), and self-reported confidence in managing their health. The form also captured social history and lifestyle behaviors to guide personalized goal setting. These baseline assessments enabled youth CHVs to tailor their coaching approaches according to each participant’s readiness, capacity, and specific areas of support required.

Youth CHVs support participants to fulfill the following objectives through the HealthStart program:

1. Learn about their chronic diseases, such as diabetes mellitus, hypertension, or hyperlipidemia, with the aid of the Health Promotion Board (HPB) booklets.
2. Learn at least one digital health app such as HealthHub or Healthy 365:
  - HealthHub is a digital platform that interfaces with medical health record systems across various public health care institutions and national source systems, such as the National Electronic Health Record and National Immunization Registry in Singapore [24].
  - Healthy 365 is a mobile app by the Health Promotion Board (HPB) Singapore that aims to encourage users to adopt a healthier lifestyle. Through the use of gamification and rewards, users are encouraged to sign up for in-app challenges and health programs to earn Healthpoints [25].
3. Enroll with a primary care provider (PCP).
4. Set a lifestyle goal and achieve it.

The participants first meet their youth CHV at a community event where health screening reports are distributed and explained by a community nurse. The youth CHVs then conduct their first session of layperson-led health coaching with the participants and build rapport via questions that cover sociodemographic factors and lifestyle aspects using tools such as a Time Wheel and a Healthy Plate (see Multimedia Appendix 1) to guide SMART goal setting.

Youth CHVs follow up with their participants longitudinally over a period of 3 months through in-person or internet-based visits (ie, video calls) every fortnight (for a minimum of six visits). They use online forms (form.sg) to track the participant’s progress through HealthStart. By fostering meaningful relationships and using motivational interviewing techniques, youth CHVs guide participants in aligning their core values and purpose with their health aspirations, facilitating deeper self-integration.

Youth CHVs also provide digital literacy training using components from a previously described program [8-10]. After teaching the basics of using a smartphone or alternative digital devices, HCVs will guide their participants to download and use health apps to achieve Objective 2 of HealthStart as described above. This is further described in Multimedia Appendix 1 (see volunteer protocol). To close the digital divide, specific guidance is provided to youth CHVs to help participants without digital devices (see Annex S7 in Multimedia Appendix 1). Youth CHVs are provided a step-by-step guide on what to do during each visit (see Table S1 in Multimedia Appendix 2).

Participants who complete the above goals may be discharged from the program before the 3-month mark. When this occurs, youth CHVs are offered the opportunity to take on additional participants. In addition, those with greater experience are encouraged to support more than 2 participants if they are willing. A total of 10%‐20% of youth CHVs return as recurrent volunteers, gradually building a pool of experienced CHVs that expands the program’s scale and capacity. HCVs and youth CHVs convene monthly to reflect on their intergenerational and service-learning experience. During these meetings, youth CHVs report on their participants’ progress, while the HCVs provide guidance and advice.

### Description of Volunteer Recruitment and Training

#### Volunteer Recruitment

Youths [26], aged 15‐35 years old, with no formal health care training or clinical experience, were recruited through volunteer portals, school outreach, and youth networks. While formal health care knowledge was not required, candidates were expected to demonstrate strong interpersonal communication skills, a willingness to learn, and a commitment to engage over a 3-month period. Selection involved completion of a pretraining knowledge quiz and participation in a full-day in-person training session. Only candidates who passed both formative assessments (content comprehension and role-play evaluations) were deployed. Those who did not meet the criteria were either redirected to other volunteering roles or offered additional training. HCVs, comprising doctors, nurses, and allied health professionals, were recruited via publicity emails and newsletters sent out to relevant professional networks.

#### Volunteer Training

HealthStart adopted a blended learning approach [27], combining self-paced online modules, a pretraining quiz, and an 8-hour in-person workshop (see Table S2 in Multimedia Appendix 2). Before training, volunteers received educational materials from the HPB on hypertension, hyperlipidemia, and diabetes mellitus, along with a protocol guidebook (see Multimedia Appendix 1). The workshop covered the Singapore health care system, chronic disease management, SMART goal setting, motivational interviewing, and the role of social determinants of health (see Multimedia Appendix 1). From the outset, volunteers were assigned to intergenerational teams to foster collaboration.

The training concluded with four standardized role-play scenarios (see Multimedia Appendix 1) simulating common participant engagement challenges. Using a standardized checklist (see Multimedia Appendix 1), HCVs assessed youth CHVs on communication skills, clinical understanding, and application of motivational interviewing techniques. Only those meeting the competency thresholds were deployed; others were offered remediation or reassigned to alternative roles. This multitiered training and evaluation process ensured consistent quality and preparedness across the volunteer cohort.

### Data Collection

Quantitative data were collected between September 2022 and June 2023. For older adult participants, data were obtained at program entry and at the 3-month endpoint using a secure FormSG digital survey administered by trained volunteers. Variables included sociodemographic, primary care follow-up status, and open-ended program feedback.

Quantitative and qualitative feedback from volunteers (CHVs and HCVs) was collected at three milestones: (1) completion of training, (2) the youth CHV’s first participant meeting, and (3) program conclusion. After-action reviews involving the organizing team and community partners were conducted at each milestone, with minutes taken and subsequently analyzed alongside volunteer and participant feedback to identify key themes.

### Data Analysis

Quantitative analyses were conducted using SPSS Statistics (version 27; IBM Corp). The primary outcome was the change in primary care follow-up status before and after participation in HealthStart. Descriptive statistics were reported with SD for calculated means and with percentages for absolute numbers. The McNemar test was used to assess paired categorical data. Missing data was handled via listwise deletion; no imputation methods were applied.

Fidelity was monitored at various levels: (1) volunteer training completion and pass rates, (2) logging of all volunteer-participant encounters on the electronic survey platform (capturing mode, duration, and coaching elements delivered), (3) cumulative visit counts per participant, and (4) participant attrition at various stages of the program. Program fidelity and outcomes will be reported in detail in a separate mixed-methods outcomes paper.

### Ethical Considerations

Ethics approval was obtained from the SingHealth Centralized Institutional Review Board (CIRB references 2022/2700 and 2023/2480). All participants provided informed consent and were given a participant information sheet. All data were anonymized and deidentified in the study. No compensation was provided to the participants.

## Results

### Program Outreach and Impact

HealthStart was implemented alongside 2 community health screening events in 2022 and 2023. Of 236 eligible individuals, 192 enrolled (79%). The mean age of the participants was 66.9 (SD 9.6) years; 47.9% (92/192) were male and 52.1% (100/192) female. Most participants were of Chinese ethnicity, had completed primary or secondary school education, resided in self-owned flats, and in 3-room public housing (see Table 1).

**Table 1.** Participant characteristics.

Characteristics	HealthStart participants (N=192)
Age (years), mean (SD)	66.9 (9.6)
Sex, n (%)	
Male	92 (47.9)
Female	100 (52.1)
Race, n (%)	
Chinese	170 (88.5)
Indian	12 (6.3)
Malay	9 (5.0)
Others	1 (0.2)
Marital status, n (%)	
Never married	31 (16.2)
Married	132 (69.1)
Divorced	7 (4.0)
Widowed	20 (10.5)
Others	1 (0.2)
Status of residential, n (%)	
Own	165 (85.9)
Lodge	9 (4.7)
Rent	18 (9.4)
Type of housing^a^, n (%)	
1-room housing	15 (7.9)
2-room public housing	11 (5.8)
3-room public housing	78 (41.1)
4-room public housing	39 (20.5)
5-room public housing	43 (22.6)
Private housing	4 (2.1)

a A total of 70% to 80% percent of Singaporeans live in public housing provided by the Singapore Government Housing Development Board (HDB) [28]. The eligibility of these public housing is means-tested, and the combined monthly income of the household must be below approximately US $8500‐$10,500. In addition, most of the 1-room public housing units are rental units and are only eligible for individuals with monthly incomes of below approximately US $1120. As such, housing type is often used as a surrogate for socioeconomic status in Singapore, with larger and private units representing higher socio-economic status [29].

Follow-up rate with primary care increased from 42.7% (82/148) preprogram to 84.5% (125/148) postprogram (*χ*^2^_1_=43; *P*<.001). In models adjusting for demographics and baseline questionnaire scores, effects were greater for older adults (odds ratio [OR] 1.8, 95% CI 1.2‐2.7) and minority ethnic groups (OR 1.6, 95% CI 1.1‐2.3). Postprogram feedback was submitted by 109 participants, including open-ended responses.

**Figure 3.**
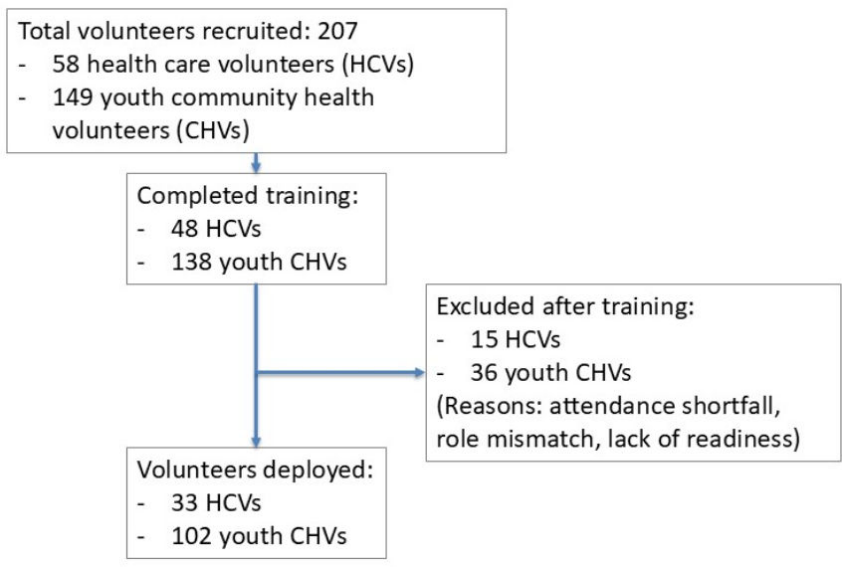
Volunteer deployment flow chart showing total recruitment of volunteers, number of volunteers excluded after training as well as final number of volunteers deployed.

Across two cycles, 207 volunteers were recruited (58 HCVs and 149 youth CHVs). Following training, 48 HCVs and 138 youth CHVs were assessed for deployment readiness. Ultimately, 33 HCVs and 102 youth CHVs were deployed. Nondeployment (15 HCVs and 36 youth CHVs) reflected nonattendance, role-expectation mismatch, or suboptimal formative assessment performance (see Multimedia Appendix 1). Figure 3 summarizes the number of volunteers at each stage of the program (recruitment, training, and deployment). The mean age of the HCVs was 37 years, 24 (72.7%) were female. A total of 26 were nurses, 6 were doctors, and the rest were allied health professionals. The mean age of the CHVs who were deployed was 24 years, and 75 (73.5%) were female. A total of 58 (56.9%) CHVs were students, and the majority were postsecondary students. In addition, 31 volunteers provided postprogram feedback.

### Program Level Reflections

#### Volunteer Competency and Selection Criteria

A multistep, competency-based selection process ensured standardized and quality layperson-led coaching. Attendance at all training components was mandatory; volunteers were required to pass a pretraining quiz and achieve competency on an HCV-rated checklist across 4 role-play scenarios. Despite completing training, 26% (36/138) of youth CHVs were not suitable for deployment in the actual program.

#### Tiering of Participant Intervention

The program involved a wide spectrum of participants, some of whom were complex with significant psychosocial issues, whereas some were already taking initiative and self-managing their conditions, thus requiring limited input from the program. To mitigate this, our program provided multiple levels of support for volunteers, including the tiering of participants. This was in keeping with what we know in the transtheoretical model, which posits that health behavior change involves progress through 6 stages of change: precontemplation, contemplation, preparation, action, maintenance, and termination [30]. Through the initial contact on the result collection day, the participants were redistributed to ensure that each group had participants of varied complexities. Each group was led by an HCV who would guide youth CHVs and address any complex questions the participants may have. Finally, an organizing committee member supervised every group of volunteers. These check-ins were crucial in helping youth CHVs refine their approach to distinct groups of participants. Furthermore, to maximize the usability of the program, a tiered model based on the participant’s stage of change may be used to tailor each volunteer’s intervention and approach.

The transtheoretical model of behavioral change has been used in this setting to gauge the stages of change and readiness for behavioral health change. In this process, participants pass through 5 stages: precontemplation, contemplation, preparation, action, and maintenance. Based on the participant’s stage of change and factors controlling the transfer between stages, youth CHVs and HCVs can better apply motivational interviewing and health coaching principles to facilitate the transition to the next stage of change. Service learning pedagogy provides the educational scaffold that enhances youth CHV’s self-efficacy and role identity. During program delivery, youth CHVs use autonomy-supportive behaviors; these behaviors, rooted in SDT, are hypothesized to enhance client autonomous motivation. SMART goal setting translates this motivation into concrete, achievable behavioral targets, thereby reinforcing competence. High-quality intergenerational interaction, as specified by contact theory, fosters relatedness and mitigates ageist stereotypes, which the transtheoretical model positions as catalysts for progression through stages of change. The combined effect of these mechanisms is expected to produce stage-matched action plans, sustained self-monitoring, and ultimately improved linkage to primary care and biometric outcomes.

### Community Level Reflections

#### Tapping on Community Assets

There are many preexisting health initiatives in the community, and an initiative such as HealthStart needs to be aligned with community health needs and existing programs to ensure sustainability and constructive collaboration. Before the launch of the program, local grassroots organizations (GROs) were engaged to obtain an understanding of community health needs and existing initiatives.

HealthStart aligned itself with the GROs’ goals of improving community health care and obtained buy-in from these stakeholders. As a result of this early alignment, the HealthStart team was able to secure practical support that directly facilitated implementation. This included logistical support such as access to venue spaces (eg, community clubs and activity centers) for resident meet-ups during result collection. Manpower support was also enhanced through on-site assistance from GRO-linked volunteers. In addition, co-branded communication materials helped to improve publicity efforts and build credibility with residents, contributing to stronger engagement during dissemination activities. Despite strong community interest, several challenges emerged in operationalizing partnerships. Space availability and volunteer scheduling also posed logistical barriers. In early cycles, inconsistent communication channels between organizing committees and community venues led to conflicting instructions. To mitigate these issues, a single-point liaison system and standardized briefing packages were introduced in later cycles, improving coordination and reducing friction.

#### Adoption of a Social Prescription Framework

To effectively improve the health outcomes of participants, the social determinants of the health of each participant need to be addressed in a deliberate manner. Social prescription aims to provide a framework to improve well-being by linking individuals to community assets [31]. During the initial cycles of HealthStart, while volunteers organically attempted to link participants to community exercise and wellness programs, there was no standardized referral pathway or tool to support this effort. This represented a key implementation gap. To address this, future iterations will incorporate a structured social prescribing referral directory and a guided conversation template. This tool will be co-developed with local partners and integrated into the volunteer protocol. Baseline assessments in the First Visit Form will be updated to capture social isolation risk and community engagement levels, allowing for more intentional linkage to social care assets during the program journey. We plan to introduce an applied version of this in HealthStart for youth CHVs in hopes that it can better guide them to address the holistic needs of the participants.

### Societal Level Reflections

#### Importance of Alignment With Population Health Policies

The success of this layperson-led health coaching program is dependent on the broader environment in which the program operates. HealthStart was launched right after HSG, allowing the program to leverage publicity, incentives for primary care enrollment, and subsidized preventive services. Furthermore, the program’s goals aligned with those of HSG, which leverages promoting health instead of just health care, as well as focusing on intergenerational bonding between participants and youth CHVs.

#### Cultivating Intergenerational Relationships

Singapore places great emphasis on the importance of intergenerational ties and intergenerational support [32,33]. Participants and volunteers reported meaningful intergenerational exchanges through HealthStart; the health screening participants shared life experiences and practical challenges with healthy living with volunteers, and the volunteers contributed their energy and fresh perspectives. Intergenerational contact is important in aging populations, as it allows society to address health-related issues through the resources provided by the younger population and intergenerational transfer of health literacy [31].

Known challenges in intergenerational programs (intergenerational conflicts, role conflict and ambiguity, inactive engagement, and difficulty in establishing meaningful connections between generations) were observed [34]. These were mitigated through the availability of HCV mentorship (eg, communication of technical terms and facilitated difficult conversations), language-matched volunteer-participants matching, proactive leadership by the organizing committee.

## Discussion

### Principal Findings

HealthStart demonstrates the feasibility of an intergenerational, layperson-led health coaching model embedded in primary care. Engaging youth CHVs with no previous health care experience (an underused resource in the community) was associated with substantial gains in primary care follow-up. Integration of SDT, SMART goals, and service-learning concepts provided a coherent framework for participant engagement and volunteer development. Participation of youth CHVs has the additional benefit of improving their adoption of healthy behaviors and diffusing health knowledge within their own communities, amplifying the impact of the program.

### Comparison With Previous Work

There has been growing interest in the potential of laypersons as health coaches [35]. Peer-led programs such as the Whole Health Action Management (WHAM) model have increased patient activation among adults with behavioral health conditions, outperforming a clinician-delivered self-management program [36]. Among college students, an 8-week peer-coaching intervention raised physical activity, positive affect, and overall well-being [37]. Smartphone-enabled coaching by nonclinicians has also lowered HbA_1c_ in type 2 diabetes [38].

HealthStart extends these peer-coaching models by adding an intergenerational component, which fosters social cohesion, empowers and expands the scope of volunteers, and introduces mentorship and guidance of these laypersons by the health care professionals. By layering structured goal setting, social prescribing, and digital health coaching onto this intergenerational platform, HealthStart addresses gaps—particularly digital literacy deficits—seldom tackled in earlier lay-coach designs.

Study protocols are being developed evaluating the comparative effectiveness of health coaching by laypersons versus health care professionals; HealthStart’s model may inform such evaluations [39,40].

### Limitations

The study has several limitations. First, the study was conducted within a specific geographical region in Singapore, which may limit its generalizability to other cultural or health care settings. Differences in population demographics and community engagement practices could influence the feasibility and effectiveness of similar programs elsewhere.

Second, the recruitment and retention of youth CHVs posed a challenge. Despite rigorous selection and training processes, a proportion of youth CHVs were found to be unsuitable for deployment, requiring additional training or reassignment to other roles. As a relatively new field, layperson-led health coaching programs are challenged by the lack of a structured framework, reimbursement, and community coalition [41].

Third, the study relied on self-reported outcomes from participants and volunteers, which may introduce response biases, particularly social desirability bias. Although objective measures such as enrollment with a primary care provider were tracked, triangulation with objective metrics was not feasible in this pilot due to limitations in data sharing agreements and system interoperability.

Finally, the absence of a control group limits causal inference. Improvement in primary care follow-up seen may reflect the effect of concurrent initiatives such as HSG.

### Future Directions

Building on initial learnings, the next phase of HealthStart will focus on improving scalability, integration, and evaluation rigor.

First, integration with national platforms under HSG will be deepened through formalized referral pathways and alignment of volunteer coaching with individualized health plans. Greater data connectivity will be pursued to enable linkage with electronic medical records and backend analytics (eg, HealthHub and Healthy365) for more robust outcome tracking.

Second, a structured social prescribing framework will be introduced. This includes enhanced baseline assessments to identify social needs (eg, isolation and financial insecurity), and a digital tool to guide volunteers in referring participants to community resources.

Third, comparative research will examine the effectiveness of youth-led versus professional-led coaching models, as well as the cultural adaptability of HealthStart across diverse settings. Digital adaptations for multilingual, underserved populations will also be explored to extend program reach and promote health equity.

Finally, to sustain scale-up, efforts will focus on reducing coordination burden via digital tools and supporting volunteer retention through micro-incentives (eg, digital badges and learning credits), structured recognition, and clearer role progression. Cost-effectiveness analyses and workforce planning will be essential to assess the long-term viability of integrating layperson-led coaching into primary care. These have to be tailored to the resource demands and contextual constraints of the specific community.

### Conclusion

HealthStart demonstrates the feasibility and promise of a structured, intergenerational, layperson-led health coaching model integrated within a primary health system. Designed with a foundation in behavior change theory, aligned with community needs, and supported by digital health tools, the program addresses key barriers to chronic disease follow-up. We identified key lessons learned in the conceptualization and implementation of the program. These insights may support efforts to replicate this model of care across diverse health systems.

## Supplementary material

10.2196/76592 Multimedia Appendix 1 Detailed HealthStart program outline and protocol.

10.2196/76592 Multimedia Appendix 2 Suggested guide for HealthStart visits and content of volunteer training.

10.2196/76592 Checklist 1 Standards for Reporting Implementation Studies (StaRI) checklist.

10.2196/76592 Checklist 2 The Template for Intervention Description and Replication (TIDieR) checklist.

## Abbreviations

CHV: community health volunteer
GRO: grassroots organization
HCV: health care volunteer
HPB: Health Promotion Board
HSG: Healthier SG
SMART: Specific, Measurable, Achievable, Realistic/Relevant, and Time-bound
