# Supplementary material for: Implementation, Challenges, and Outlook of an Intergenerational, Layperson-led, Health Coaching Program (HealthStart): A Pilot Case Study
Source: JMIR Form Res. 2025 Sep 22;9:e76592. doi: 10.2196/76592 (PMC12453286; doi:10.2196/76592)
Supplement: Multimedia Appendix 1 [file formative-v9-e76592-s001.docx]

- *The manuscript uses the term youth community health volunteers (youth CHVs), and this is interchangeable with the terms non-healthcare volunteers (nHCVs) used in the appendix.*

**Appendix S1: Materials used during first health coaching session**

**
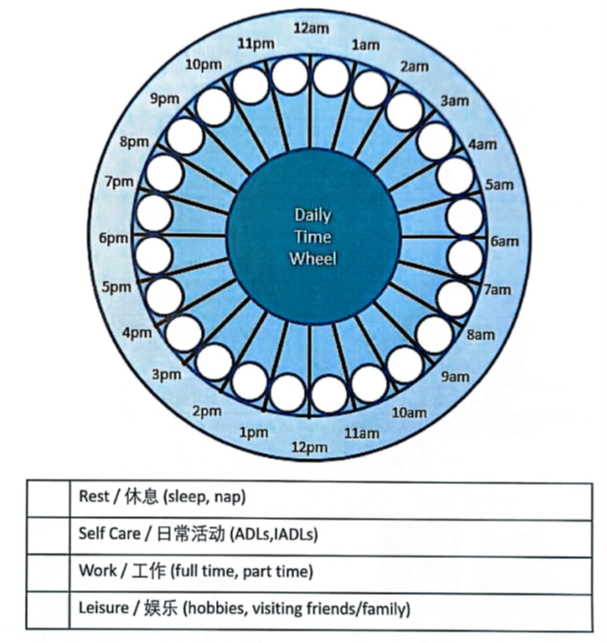
**

Figure S1: Time Wheel

**
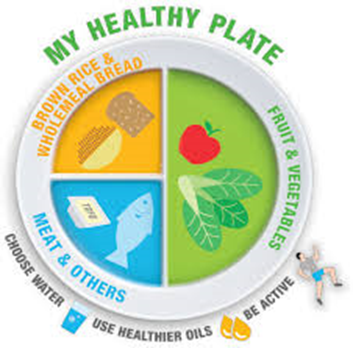
**

Figure S2: Healthy Plate

**Appendix S1: Volunteer protocol guidebook**


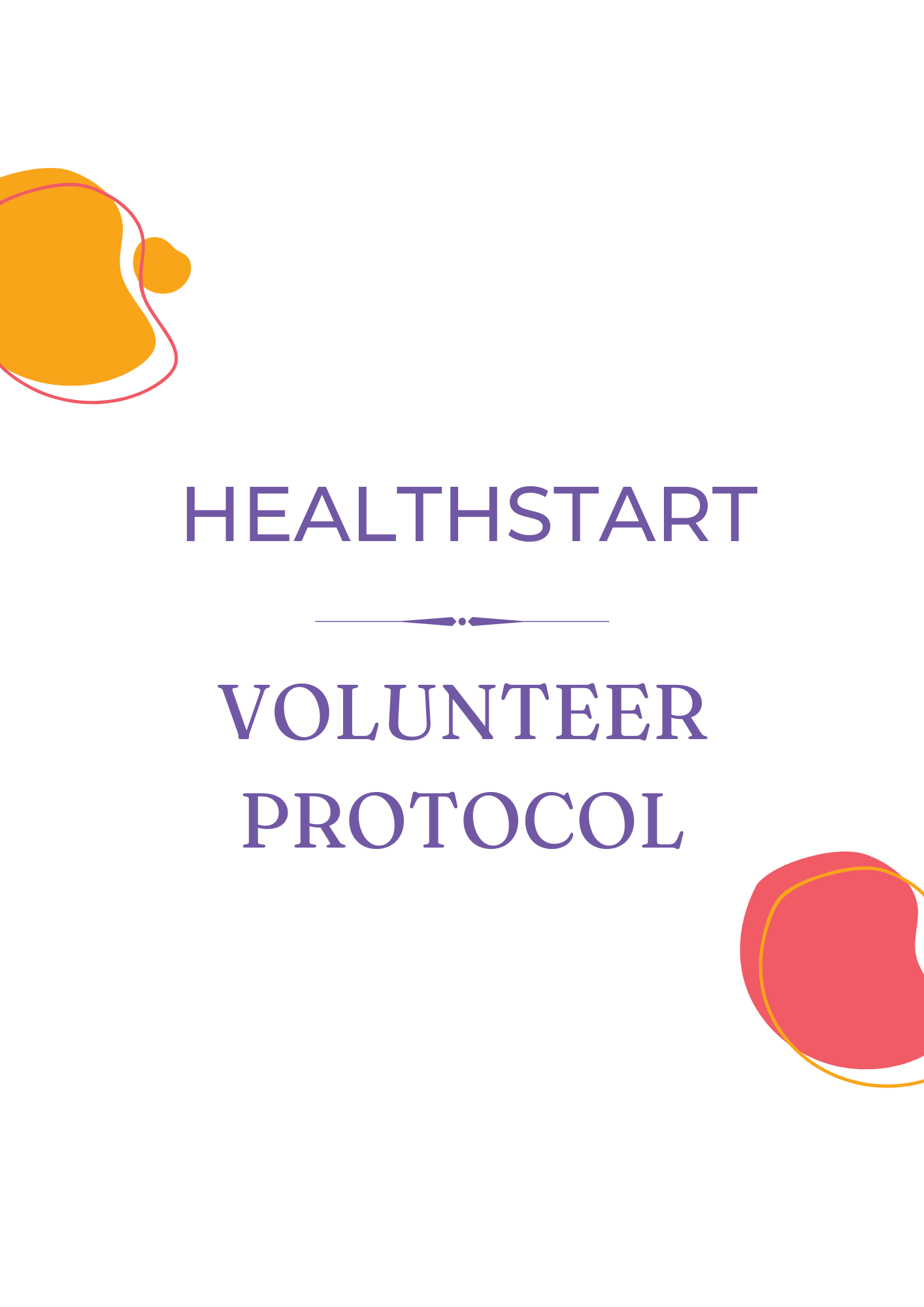

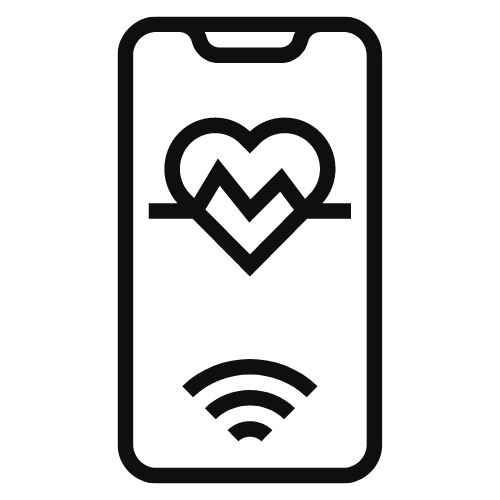


**Contents**

[**What is HealthStart? 3**](#_gjdgxs)

[**Volunteer Training 4**](#_30j0zll)

[Pre-Training E-learning Materials 4](#_1fob9te)

[HealthStart Physical Volunteer Training Session 4](#_3znysh7)

[Volunteer Readiness Checklist 5](#_2et92p0)

[**Overview of HealthStart 6**](#_tyjcwt)

[Flow of events 6](#_3dy6vkm)

[Group structure 6](#_1t3h5sf)

[**Health Screening Results Collection Day 7**](#_4d34og8)

[Process Flow on Results Collection Day (for residents collecting in person) 7](#_2s8eyo1)

[Process Flow for Results Disclosure (for residents unable to collect in person) 7](#_17dp8vu)

[**Health Coaching by Volunteers Checklist/Guide 8**](#_3rdcrjn)

[For All residents (with chronic condition/borderline high results) 9](#_26in1rg)

[For Residents with Borderline High or High Cholesterol 12](#_lnxbz9)

[For Residents with Borderline High or High Blood Pressure 14](#_35nkun2)

[For Residents with Borderline High or High Blood Sugar 16](#_1ksv4uv)

[**Role of Healthcare Volunteers 18**](#_44sinio)

[**Frequently Asked Questions/Potential Scenarios Encountered 19**](#_2jxsxqh)

[**Escalation of Challenges/Feedback/Ideas 20**](#_z337ya)

[**Annexes 21**](#_3j2qqm3)

[Annex S1: Pre-Training Materials 21](#_1y810tw)

[Annex S2: Health counseling modules/goal setting 21](#_4i7ojhp)

[Annex S3: Health-related phone apps 26](#_2xcytpi)

[Annex S4: Online health resources 27](#_1ci93xb)

[Annex S5: Safety Protocol 28](#_3whwml4)

[Annex S6: SingPass Application 29](#_2bn6wsx)

[Annex S7: Digital Device and Plans Available 29](#_qsh70q)

[Annex S8: Summary of forms to fill and other relevant documents 31](#_3as4poj)

# **What is HealthStart?**

HealthStart is a volunteer-led health coaching programme targeting residents with newly diagnosed or uncontrolled chronic diseases as well as residents with borderline high screening results identified at health screening events. Volunteers are assigned to these residents with the aim to promote health and wellbeing for these residents through adoption of digital technology.

The at the end of the HealthStart programme is for the resident to:

**Go through** the Health Promotion Board (HPB) booklets on the condition the resident was newly diagnosed with

**Pick up** at least 1 digital health app i.e. HealthHub

**Set** at least 1 SMART goal and assist the resident in **achieving** it

**Ensure** the resident follows up with a Doctor

This can be done via Healthier SG enrollment for the resident to visit their nearby GP https://www.healthiersg.gov.sg/enrolment/guide/

**Fill** up the FormSG form after every visit (be it physical/video/phone call)

**Bonus goal:** advise the resident to undergo further age appropriate health screenings such as cancer screening, adult vaccinations

5 + 1 Bonus Goals

This volunteer protocol contains references and information to aid in your volunteering journey and best value add to our residents. It will be your guide to achieve the goals set out for this HealthStart programme.

It contains information on:

- Chronic diseases - to **educate** the residents about their disease condition to complete health counseling.
- Communication skills i.e. motivational interview - to **encourage** the resident to become an active participant in their own health through setting lifestyle goals to achieve 1 lifestyle modification goal
- Health applications i.e. Healthy365, HealthHub - to introduce health applications to the resident to **engage** them in health management
- Singapore healthcare landscape - to **empower** and guide the residents in making an appointment at the appropriate and proper healthcare institution to guide seniors in initiating the first polyclinic/GP visit

#

# **Volunteer Training**

The volunteer protocol contains information for both healthcare volunteers (doctors and registered nurses) and non-healthcare volunteers (including enrolled nurses, medical and nursing students). We strongly encourage healthcare volunteers to look through all the materials (training materials and overview of HealthStart) so as to better mentor the non-healthcare volunteers assigned to you. The main role and responsibilities as a healthcare volunteer can be found in [this section](#_44sinio).

## **Pre-Training E-learning Materials**

These materials are compulsory for non-healthcare volunteers (including enrolled nurses, medical and nursing students) to help build confidence when speaking to the residents about these topics.


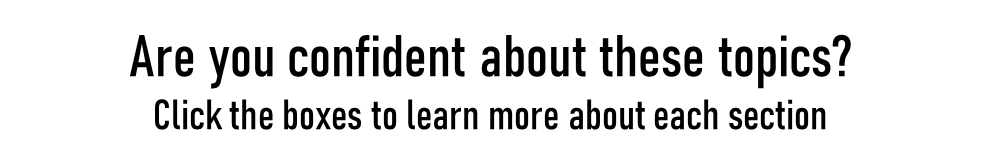


[
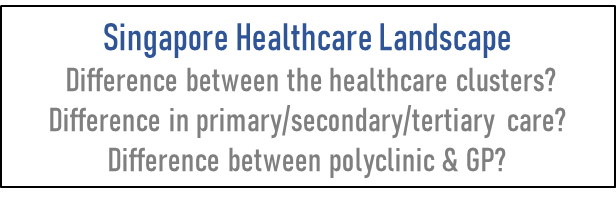
](https://docs.google.com/document/d/17bIiG4YUx0hZfGgegHmdZxETVgdnhwdnKEsNCGOm5Hk/edit#bookmark=id.u20vpklbnlp)
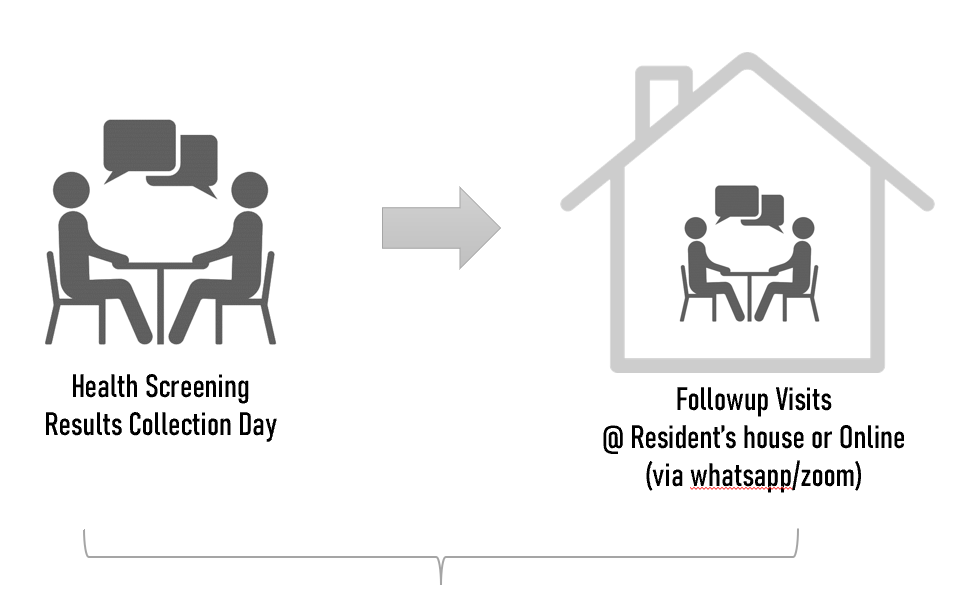


[
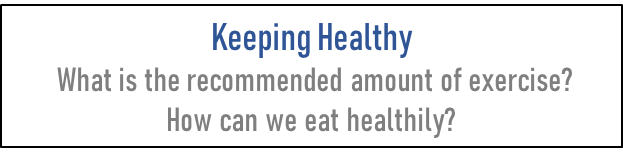
](https://docs.google.com/document/d/17bIiG4YUx0hZfGgegHmdZxETVgdnhwdnKEsNCGOm5Hk/edit#bookmark=id.2xg15kxgi2rt)

[
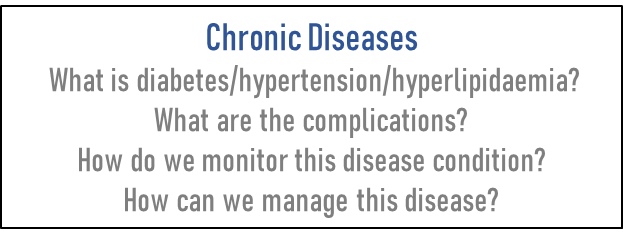
](https://docs.google.com/document/d/17bIiG4YUx0hZfGgegHmdZxETVgdnhwdnKEsNCGOm5Hk/edit#bookmark=id.8lk3i4yr002)

[
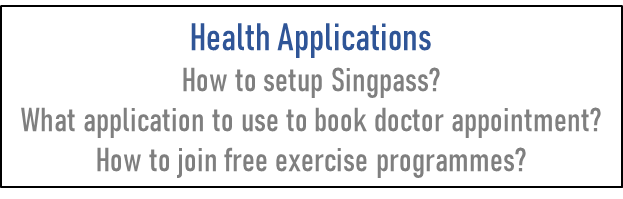
](https://docs.google.com/document/d/17bIiG4YUx0hZfGgegHmdZxETVgdnhwdnKEsNCGOm5Hk/edit#bookmark=id.f6y013t1d9hd)[
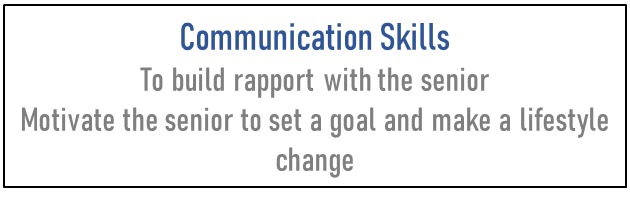
](https://docs.google.com/document/d/17bIiG4YUx0hZfGgegHmdZxETVgdnhwdnKEsNCGOm5Hk/edit#bookmark=id.bd02m6s0kmgs)
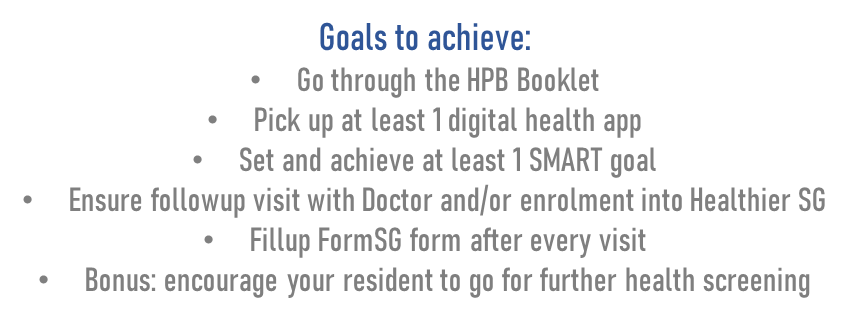


##

## **HealthStart Physical Volunteer Training Session**

Attend the volunteer training session to learn how to apply all the information and skills learned in the e-learning materials. Do remember to bring along your **hardcopy signed volunteer participation form** and have your phone fully charged with all the [required apps](#3o7alnk) downloaded.

##

## **Volunteer Readiness Checklist**

If you are familiar with all the content in this checklist, you are ready to be assigned a resident 🙂


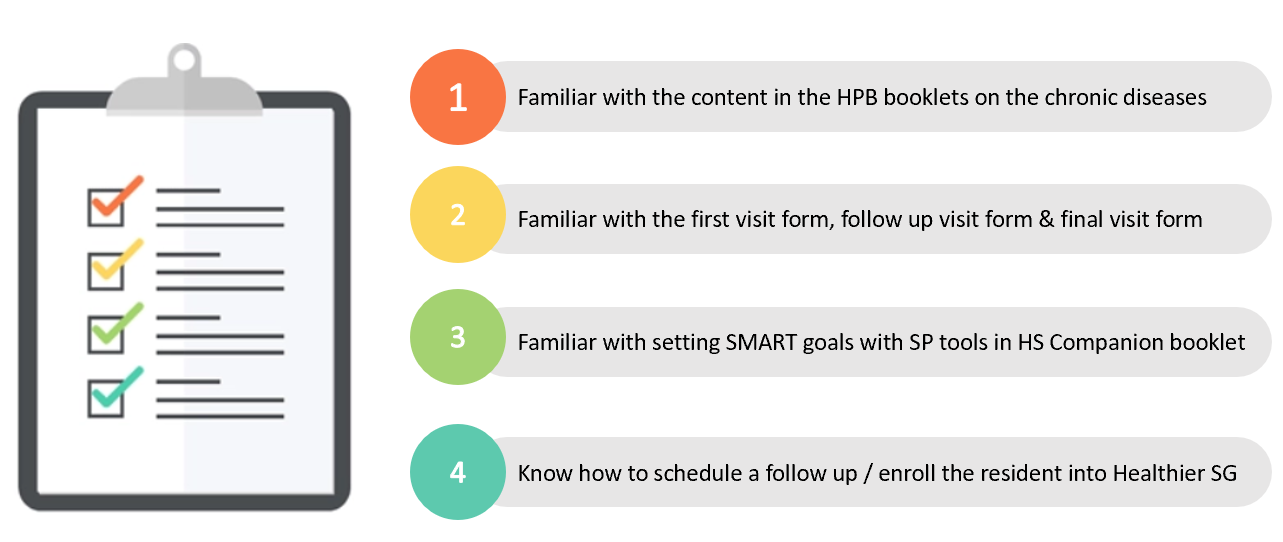


##

# **Overview of HealthStart**

## **Flow of events**


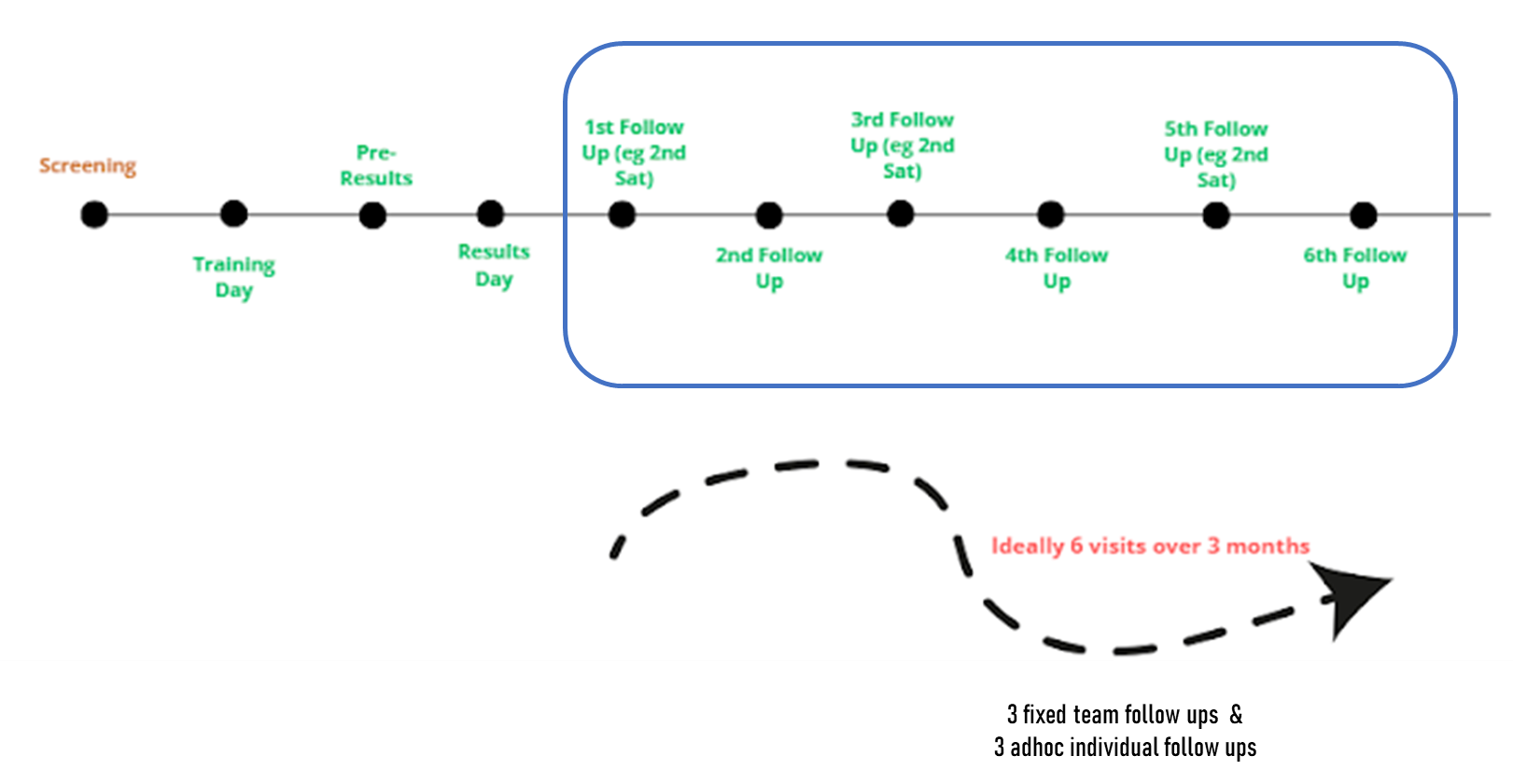


## **Group structure**

- 1 group will consist of 1 healthcare volunteer and 4-6 non-healthcare volunteers. Volunteers who are 18 years old or under will be in pairs.
- Each group will be responsible for 8 residents (up to 10 residents)
- Shared responsibility of 8-12 residents among the 4-6 non-healthcare volunteers


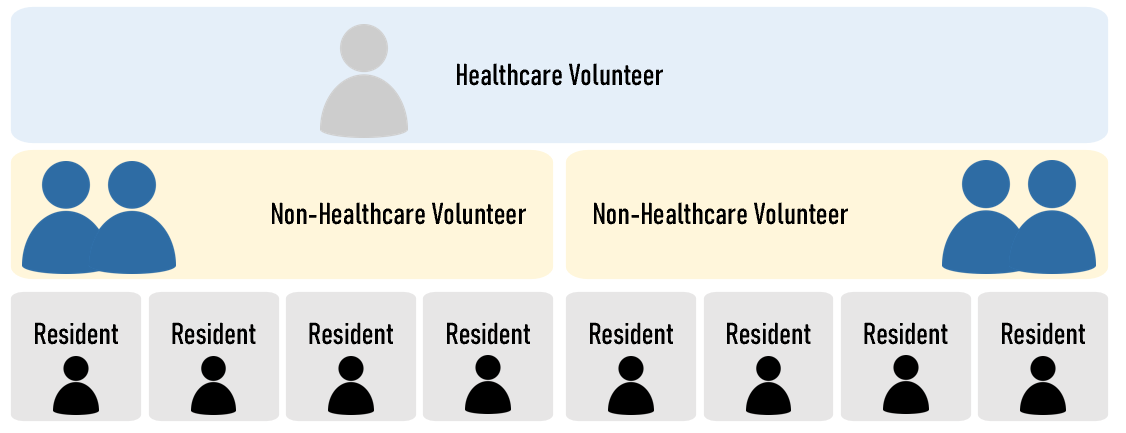


# **Health Screening Results Collection Day**

|  | Attire | - TriGen T-shirt OR YCS T-shirt (given on training day), long pants, covered shoes and volunteer lanyard tag |
| --- | --- | --- |
|  | What to bring | - Small sling bag if required for necessities - handphone, wallet   - May not have an area to place bags for safekeeping - Laptop/ tablet for health coaching |

## **Process Flow on Results Collection Day (for residents collecting in person)**

For residents who collect their health screening results in person:


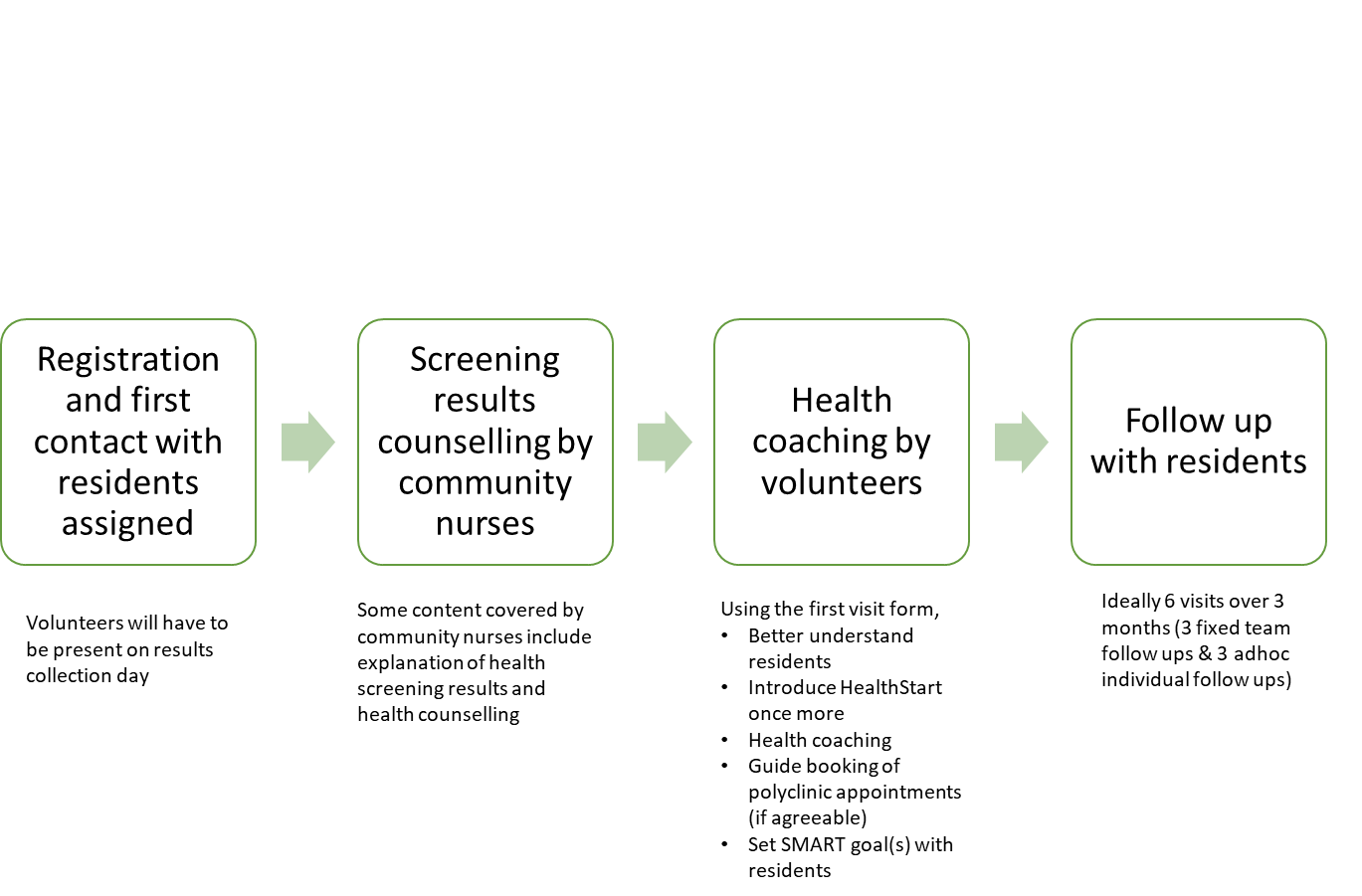


## **Process Flow for Results Disclosure (for residents unable to collect in person)**

For residents who are unable to attend results collection in person:


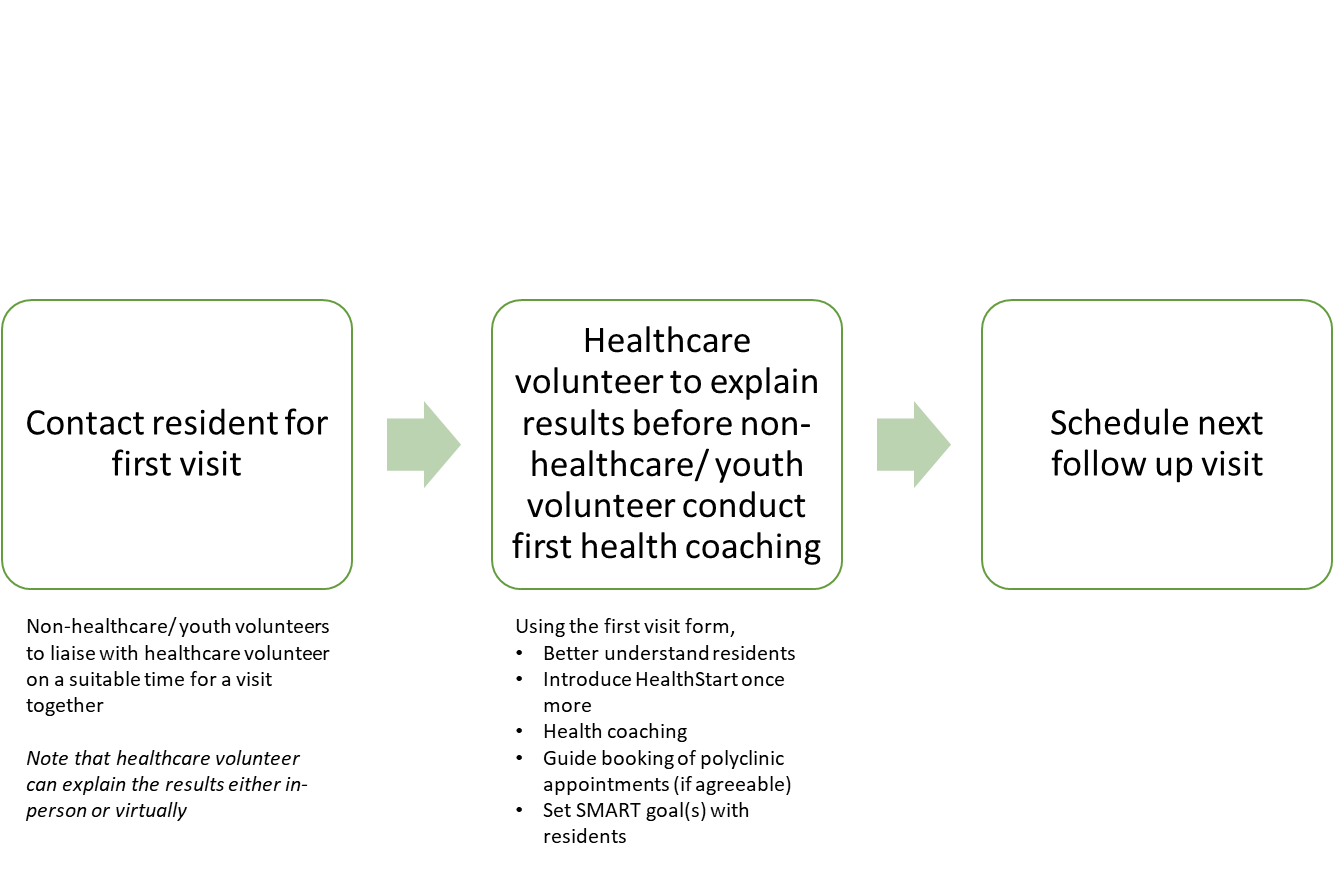


###

# **Health Coaching by Volunteers Checklist/Guide**

Select the box that applies to your resident.

This “All Residents” segment will guide you on the steps to take during results collection day, follow up sessions as well as final visit for the resident assigned to you.

[
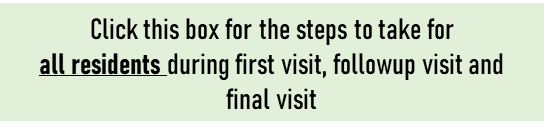
](#_26in1rg)

The following medical condition specific segment will guide you on questions to ask tailored to that medical condition. Through these questions you can find out more about the lifestyle of the resident and determine where would be a good area to target/introduce lifestyle modifications. This will also aid in setting/modifying the SMART goal such that it is more tailored to the resident. The different segments also provides many resources catered to the medical condition.

[
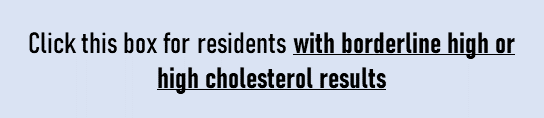
](#_lnxbz9)

[
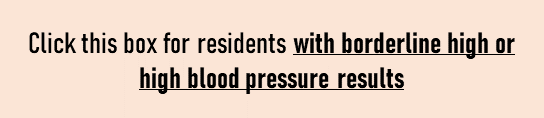
](#_35nkun2)

[
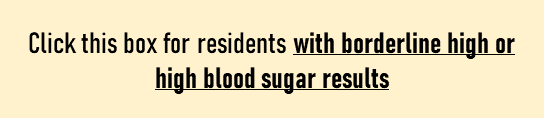
](#_1ksv4uv)

## **For All residents (with chronic condition/borderline high results)**

| **No.** | **Action** | **Done?** |
| --- | --- | --- |
| **Section 0: Prior to Results Collection Day** | | |
| 1 | Discuss with your group the dates in which your team plans to do a team follow up and note down the dates. Plan the dates for the follow up 6 visits - total of 3 team visits and 3 adhoc individual visits. |  |
| 2 | Ensure familiarity with disease conditions (hypertension, hyperlipidaemia, diabetes) and the lifestyle modifications/social prescribing that can help with these disease conditions. |  |
| 3 | Ensure familiarity with the [first visit form](https://for.sg/hs3firstvisit) prior to results collection and /or disclosure day. |  |
| 4 | Ensure familiarity in scheduling an appointment on Healthhub / enrolling the resident into Healthier SG |  |
| **Section 1a: Results Collection Day (Residents collect in person) (1 Session)** | | |
| 1 | Ensure that the resident is aware that they have abnormal health screening results (high blood glucose/high blood pressure/high cholesterol) or have a borderline high result as explained and counseled by the community nurse |  |
| 2 | Complete [first visit form](https://for.sg/hs3firstvisit). The first visit form takes approximately 30 mins to complete and allows you to understand the resident’s eHealth literacy, health ownership, knowledge of chronic conditions and social history. Set a SMART goal with the resident as well with the first visit form as guidance. |  |
| 3 | Introduce HealthStart to the resident and sign the media consent form. |  |
| 4 | Pass the HealthStart Booklet “My Companion Guide to a Healthier Lifestyle (HealthStart)” to the resident and complete Page 3 of the booklet “Visit 1/Results Collection Day”. Explain to them the “ABC Card” and that this booklet will be used through the HealthStart Journey. |  |
| 5 | Exchange numbers with the resident and inform the resident of the follow up date with the resident. Explain to residents subsequent follow ups can be done with phone calls, [Zoom](https://www.imda.gov.sg/seniorsgodigital/-/media/Seniors-Go-Digital/PDF/pdf5/Zoom_English.pdf)/[WhatsApp video calls](https://faq.whatsapp.com/785056755306362/?locale=en_US)/physical visits. |  |
| 6 | Ask the resident if they have any concerns. Highlight to your healthcare volunteer if the residents seem to be highly distressed about their results. |  |
| **Section 1b: Results Disclosure Day (Residents unable to collect in person) (1 Session)** | | |
| 1 | Contact the resident and schedule a physical visit with the resident. Ensure the healthcare volunteer in your group is able to attend the visit (physically or virtually). Do also ensure that the resident has received his/ her health screening report prior to the visit. |  |
| 2 | On the scheduled date, the healthcare volunteer is to disclose the health screening results to the resident. Explain how the results can be interpreted (abnormal/ borderline high), complications of the disease condition, and briefly how it can be controlled. |  |
| 3 | After results have been disclosed by the healthcare volunteer, non-healthcare volunteers complete the [first visit form](https://for.sg/hs3firstvisit). The first visit form takes approximately 30 mins to complete and allows you to understand the resident’s eHealth literacy, health ownership, knowledge of chronic conditions and social history. Set a SMART goal with the resident as well with the first visit form as guidance. |  |
| 4 | Introduce HealthStart to the resident and sign the media consent form. |  |
| 5 | Pass the HealthStart Booklet “My Companion Guide to a Healthier Lifestyle (HealthStart)” to the resident and complete Page 3 of the booklet “Visit 1/Results Collection Day”. Explain to them the “ABC Card” and that this booklet will be used through the HealthStart Journey. |  |
| 6 | Inform the resident of the follow up date with the resident. Explain to residents subsequent follow ups can be done with phone calls, [Zoom](https://www.imda.gov.sg/seniorsgodigital/-/media/Seniors-Go-Digital/PDF/pdf5/Zoom_English.pdf)/[WhatsApp video calls](https://faq.whatsapp.com/785056755306362/?locale=en_US)/physical visits. |  |
| 7 | Ask the resident if they have any concerns. Highlight to your healthcare volunteer if the residents seem to be highly distressed about their results. |  |
| **Section 2: Followup Visit (5 sessions)** | | |
| 1 | **For Group follow ups:** All members of the group to gather at a meeting point, run through action plan and raise any concerns before dispersing to visit their residents.  **For adhoc/individual follow ups**: Inform your healthcare volunteer the date and time of physical visit or virtual call one day before the visit date. |  |
| 2 | **Doctor Follow up visit:** Check if the resident has seen the doctor/ set an appointment since diagnosed with abnormal results. Enroll the resident into Healthier SG if not yet enrolled.  ([https://www.healthiersg.gov.sg/enrolment/guide/)](https://www.healthiersg.gov.sg/enrolment/guide/) |  |
| 3 | **SMART goal:** Based on the disease condition, proceed to the relevant table in the respective section for lifestyle modifications that can be performed to better manage the disease condition. There are guiding questions to ask your resident to aid in setting a smart goal as well as to check their progress with regards to their SMART goal. The relevant sections have useful resources to aid with your health coaching.   - [Borderline high or high cholesterol](#_lnxbz9) - [Borderline high or high blood pressure](#_35nkun2) - [Borderline high or high blood sugar](#_1ksv4uv) |  |
| 4 | **SMART goal:** Complete the corresponding visit page (visit 2-6) in HealthStart Booklet “My Companion Guide to a Healthier Lifestyle (HealthStart)”. Set a new SMART goal with the resident if the previous SMART goal has been achieved (refer to [Annex B](#_4i7ojhp) on potential SMART Goals). |  |
| 5 | **Digital:** Check if resident is keen to learn digital apps (possible apps include Singpass, HealthHub App, Healthy365)  Refer to [Annex G](#_qsh70q) if the resident does not have a digital device and refer to [Annex F](#_2bn6wsx) for how to use the pin mailer to apply for Singpass.  Teach the resident how to track appointments and results, check for health screening eligibility, and make appointments via HealthHub. Do also share with residents on how they can sign up for active lifestyle events and participate in Eat, Drink, Shop programme to earn rewards on Healthy365 |  |
| 6 | **Optional:** advise and encourage your resident to go for further health screening i.e. age appropriate cancer screening, adult vaccinations etc |  |
| 7 | If resident is **keen** for further follow ups during the visit that you have with him/ her: fill up [follow visit form](https://for.sg/hs3followupvisit) after each follow up visit.  If resident is **not keen** for further follow ups during the visit that you have with him/ her: fill up [final visit form](https://for.sg/hs3finalvisit). |  |
| 8 | Track progress of the resident on ABC card and encourage the resident to collect all stamps to receive a goodie bag. Remind the resident of the next visit date. |  |
| 9 | **For group followups:** Gather at the end of the visits for a meal/drink. Debrief with the healthcare volunteer as a group and ask for advice from each other and the healthcare volunteer on how to better encourage the resident further to meet the HealthStart Goals.  **For adhoc/individual followup:** Message your healthcare volunteer or even your group a summary of the visit and raise any questions/concerns to your healthcare volunteer/group members if needed. Ask for advice from each other and/or the healthcare volunteer on how to better encourage the resident further to meet the HealthStart Goals. |  |
| **Section 3: Final Visit and HealthStart Goals to Complete (1 session)** | | |
| 1 | Fill up final visit form. |  |
| 2 | Complete the final visit page (visit 7) in HealthStart Booklet “My Companion Guide to a Healthier Lifestyle (HealthStart)”. |  |
| 3 | Ensure all the following HealthStart goals are completed: |  |
|  | 1. Ensure Health Promotion Board (HPB) booklet(s) on the disease condition(s) have been completed |  |
|  | 1. Achieved at least 1 SMART goal set |  |
|  | 1. Taught at least 1 digital health app e.g. HealthHub, Healthy365, Healthbuddy |  |
|  | 1. Resident followed up with a polyclinic/GP on his/her newly diagnosed condition and/or enrolled into Healthier SG. |  |
|  | 1. Ensure all follow up visit and final visit FormSG forms are filled |  |
|  | Bonus: advised and encouraged resident to go for further health screening i.e. age appropriate cancer screening, adult vaccinations etc |  |

## **For Residents with Borderline High or High Cholesterol**

| Diet | | |
| --- | --- | --- |
|  | Ask what the resident consumes for breakfast, lunch, dinner, drinks and snacks. Ask about portions and how the food is prepared (e.g. deep fried, boiled, steamed etc). |  |
|  | Refer to page 6 of “[Cholesterol Matters](https://drive.google.com/file/d/1jXI6OFUtibmBf1RBu2tDoS7QTC2ff-w7/view?usp=sharing)” for different types of fats in our diet |  |
|  | Refer to page 7 of “[Cholesterol Matters](https://drive.google.com/file/d/1jXI6OFUtibmBf1RBu2tDoS7QTC2ff-w7/view?usp=sharing)” commonly asked questions for cholesterol |  |
|  | Refer to My Healthy Plate for further dietary advice  <https://www.healthhub.sg/live-healthy/1332/plan-your-meals-with-my-healthy-plate> |  |
|  | Provide the following resources for health recipes for the residents:  Nutritional Guide:  [https://www.healthhub.sg/sites/assets/Assets/Programs/resident-health-nutrition/pdf/Nutrition_Guide-The_Recipe_For_Healthy_Ageing_(English).pdf](https://www.healthhub.sg/sites/assets/Assets/Programs/senior-health-nutrition/pdf/Nutrition_Guide-The_Recipe_For_Healthy_Ageing_(English).pdf)  Recipe Book:  <https://www.healthhub.sg/sites/assets/Assets/Programs/resident-health-nutrition/pdf/Recipe_Book-The_Recipe_For_Healthy_Ageing_(English).pdf>  My Healthy Plate:  <https://www.healthhub.sg/programmes/55/my-healthy-plate>  Healthy Recipes:  <https://www.healthhub.sg/programmes/54/recipes> |  |
|  | Encourage residents to also keep a food diary and use a tracking app such as My Fitness Pal |  |
|  | Set or modify the SMART Goal and follow up in subsequent visits (refer to [Annex B](#_4i7ojhp) on potential SMART Goals) |  |
| Exercise | | |
|  | Ask what the resident does for exercise in terms of:   1. Modality of exercise – walking/running/gym classes 2. Duration per session 3. Frequency per week |  |
|  | Counsel on adequate exercise – 30 mins each time, 5 times a week.  Counsel on adequate intensity – to break into light sweat |  |
|  | Potential areas of incorporating exercises:   1. Daily walks around the park 2. Sign up for exercises at AAC 3. Sign up for exercises under ActiveSG 4. Sign up for exercises under [CALM](https://members.myactivesg.com/programmes/result?keyword=age+related&activity_filter=&venue_filter=&start_filter=&end_filter=&search=Submit&__hstc=138112879.41c53ca9b61722f3326e694e0023eee8.1656557020350.1656557020350.1656557020350.1&__hssc=138112879.1.1656557020350&__hsfp=143745727&hsCtaTracking=70cf79a7-10b4-48da-a695-32f73409da57%7Cb4367db1-9d64-48e5-a24a-f8548525a850) |  |
|  | Home exercises that can be done with videos to follow:  Health Promotion Board Exercise Programmes:  <https://youtu.be/4UCkKDlXYk4> (English)  <https://youtu.be/1Z_VH-uGKIM> (Mandarin)  <https://youtu.be/P_UQdfd0jmE> (Malay)  <https://youtu.be/XDWzcpmAxRU> (Tamil)  Anytime, Anywhere Workout <https://youtu.be/QA6X7VJklG4> |  |
|  | Set or modify the SMART Goal and follow up on subsequent visits (refer to [Annex B](#_4i7ojhp) on potential SMART Goals) |  |
| Weight loss | | |
|  | Encourage residents with BMI 23 and above to get a weighing machine |  |
|  | Monitor weight every month |  |
|  | To set SMART goals with resident related to diet modifications and exercise which will help with weight loss |  |
| Smoking Cessation | | |
|  | For residents who smoke, encourage them to quit smoking using I Quit Programme (<https://www.healthhub.sg/programmes/88/IQuit>) |  |
|  | Set or modify the SMART Goal and follow up in subsequent visits (refer to [Annex B](#_4i7ojhp) on potential SMART Goals) |  |

##

## **For Residents with Borderline High or High Blood Pressure**

| Diet | | |
| --- | --- | --- |
|  | Ask what the resident consumes for breakfast, lunch, dinner, drinks and snacks. Ask about portions and how the food is prepared (e.g. deep fried, boiled, steamed etc). |  |
|  | Advise on low salt diet. Refer to page 9 – 12 of [Your Guide to Lowering Blood Pressure](https://drive.google.com/file/d/1Z0ixG6Lrul1HBXNzVHCdPGuRIRSSMM0E/view?usp=sharing) for areas to focus on |  |
|  | Refer to My Healthy Plate for further dietary advice  <https://www.healthhub.sg/live-healthy/1332/plan-your-meals-with-my-healthy-plate> |  |
|  | Provide the following resources for health recipes for the residents:  Nutritional Guide:  [https://www.healthhub.sg/sites/assets/Assets/Programs/resident-health-nutrition/pdf/Nutrition_Guide-The_Recipe_For_Healthy_Ageing_(English).pdf](https://www.healthhub.sg/sites/assets/Assets/Programs/senior-health-nutrition/pdf/Nutrition_Guide-The_Recipe_For_Healthy_Ageing_(English).pdf)  Recipe Book:  <https://www.healthhub.sg/sites/assets/Assets/Programs/resident-health-nutrition/pdf/Recipe_Book-The_Recipe_For_Healthy_Ageing_(English).pdf>  My Healthy Plate:  <https://www.healthhub.sg/programmes/55/my-healthy-plate>  Healthy Recipes:  <https://www.healthhub.sg/programmes/54/recipes> |  |
|  | Encourage residents to also keep a food diary and use a tracking app such as My Fitness Pal |  |
|  | Set or modify the SMART Goal and follow up in subsequent visits (refer to [Annex B](#_4i7ojhp) on potential SMART Goals) |  |
| Exercise | | |
|  | Ask what the resident does for exercise in terms of:   1. Modality of exercise – walking/running/gym classes 2. Duration per session 3. Frequency per week |  |
|  | Counsel on adequate exercise – 30 mins each time, 5 times a week.  Counsel on adequate intensity – to break into light sweat |  |
|  | Potential areas of incorporating exercises:   1. Daily walks around the park 2. Sign up for exercises at AAC 3. Sign up for exercises under ActiveSG 4. Sign up for exercises under [CALM](https://members.myactivesg.com/programmes/result?keyword=age+related&activity_filter=&venue_filter=&start_filter=&end_filter=&search=Submit&__hstc=138112879.41c53ca9b61722f3326e694e0023eee8.1656557020350.1656557020350.1656557020350.1&__hssc=138112879.1.1656557020350&__hsfp=143745727&hsCtaTracking=70cf79a7-10b4-48da-a695-32f73409da57%7Cb4367db1-9d64-48e5-a24a-f8548525a850) |  |
|  | Home exercises that can be done with videos to follow:  Health Promotion Board Exercise Programmes:  <https://youtu.be/4UCkKDlXYk4> (English)  <https://youtu.be/1Z_VH-uGKIM> (Mandarin)  <https://youtu.be/P_UQdfd0jmE> (Malay)  <https://youtu.be/XDWzcpmAxRU> (Tamil)  Anytime, Anywhere Workout<https://youtu.be/QA6X7VJklG4> |  |
|  | Set or modify the SMART Goal and follow up in subsequent visits (refer to [Annex B](#_4i7ojhp) on potential SMART Goals) |  |
| Self-Monitoring of Blood Pressure | | |
|  | If resident is keen to self monitor their blood pressure:   - Consider purchasing a blood pressure monitor at Watsons/Guardian/Unity |  |
|  | Teach residents how to use blood pressure machine   - To take at the same time everyday - To take 1h after taking blood pressure medications - Not to take BP immediately after exercise/rushing/eating |  |
|  | Show residents how to record blood pressure readings to keep a blood pressure diary. Refer to page 8 of [Your Guide to Lowering Blood Pressure](https://drive.google.com/file/d/1Z0ixG6Lrul1HBXNzVHCdPGuRIRSSMM0E/view?usp=sharing) for blood pressure targets |  |
|  | Set or modify the SMART Goal and follow up in subsequent visits (refer to [Annex B](#_4i7ojhp) on potential SMART Goals). During follow up visits, ensure compliance to blood pressure monitoring. |  |
| Smoking Cessation | | |
|  | For residents who smoke, encourage them to quit smoking using I Quit Programme (<https://www.healthhub.sg/programmes/88/IQuit>) |  |
|  | Set or modify the SMART Goal and follow up in subsequent visits (refer to [Annex B](#_4i7ojhp) on potential SMART Goals) |  |
| Weight loss | | |
|  | Encourage residents with BMI 23 and above to get a weighing machine |  |
|  | Monitor weight every month |  |
|  | To set SMART goals with resident related to diet modifications and exercise which will help with weight loss |  |

##

## **For Residents with Borderline High or High Blood Sugar**

| Diet | | |
| --- | --- | --- |
|  | Ask what the resident consumes for breakfast, lunch, dinner, drinks, snacks, desserts. Ask about portions and how the food is prepared (e.g. deep fried, boiled, steamed etc). |  |
|  | Refer to page 13 of [Living with Diabetes](https://drive.google.com/file/d/1in3pJ3-cD0x_bgearxYB4tHVGTA5O44h/view?usp=sharing) for My Healthy Plate |  |
|  | Refer to pages 8-19 of [Living with Diabetes](https://drive.google.com/file/d/1in3pJ3-cD0x_bgearxYB4tHVGTA5O44h/view?usp=sharing) for sample meal plans and healthier dietary choices |  |
|  | Provide the following resources for health recipes for the residents:  Nutritional Guide:  [https://www.healthhub.sg/sites/assets/Assets/Programs/resident-health-nutrition/pdf/Nutrition_Guide-The_Recipe_For_Healthy_Ageing_(English).pdf](https://www.healthhub.sg/sites/assets/Assets/Programs/senior-health-nutrition/pdf/Nutrition_Guide-The_Recipe_For_Healthy_Ageing_(English).pdf)  Recipe Book:  <https://www.healthhub.sg/sites/assets/Assets/Programs/resident-health-nutrition/pdf/Recipe_Book-The_Recipe_For_Healthy_Ageing_(English).pdf>  My Healthy Plate:  <https://www.healthhub.sg/programmes/55/my-healthy-plate>  Healthy Recipes:  <https://www.healthhub.sg/programmes/54/recipes> |  |
|  | Encourage residents to also keep a food diary and use a tracking app such as My Fitness Pal |  |
|  | Set or modify the SMART Goal and follow up in subsequent visits (refer to [Annex B](#_4i7ojhp) on potential SMART Goals) |  |
| Exercise | | |
|  | Ask what the resident does for exercise in terms of:   1. Modality of exercise – walking/running/gym classes 2. Duration per session 3. Frequency per week |  |
|  | Counsel on adequate exercise – 30 mins each time, 5 times a week.  Counsel on adequate intensity – to break into light sweat |  |
|  | Potential areas of incorporating exercises:   1. Daily walks around the park 2. Sign up for exercises at AAC 3. Sign up for exercises under ActiveSG 4. Sign up for exercises under [CALM](https://members.myactivesg.com/programmes/result?keyword=age+related&activity_filter=&venue_filter=&start_filter=&end_filter=&search=Submit&__hstc=138112879.41c53ca9b61722f3326e694e0023eee8.1656557020350.1656557020350.1656557020350.1&__hssc=138112879.1.1656557020350&__hsfp=143745727&hsCtaTracking=70cf79a7-10b4-48da-a695-32f73409da57%7Cb4367db1-9d64-48e5-a24a-f8548525a850) |  |
|  | Home exercises that can be done with videos to follow:  Health Promotion Board Exercise Programmes:  <https://youtu.be/4UCkKDlXYk4> (English)  <https://youtu.be/1Z_VH-uGKIM> (Mandarin)  <https://youtu.be/P_UQdfd0jmE> (Malay)  <https://youtu.be/XDWzcpmAxRU> (Tamil)  Anytime, Anywhere Workout<https://youtu.be/QA6X7VJklG4> |  |
|  | Set or modify the SMART Goal and follow up in subsequent visits (refer to [Annex B](#_4i7ojhp) on potential SMART Goals) |  |
| Self-Monitoring of Sugar Control / Uncontrolled Diabetes Complications | | |
|  | If resident is keen to self monitor their blood sugar:   - Consider purchasing a glucometer at Watsons/Guardian/Unity/Mustafa |  |
|  | To arrange with healthcare volunteer to teach residents how to use. |  |
|  | Explain to resident regarding need for eye and foot screen |  |
|  | Refer to pages 23 – 24 on [Living with Diabetes](https://drive.google.com/file/d/1in3pJ3-cD0x_bgearxYB4tHVGTA5O44h/view?usp=sharing) for foot care advice |  |
|  | To check if residents have made an appointment for eye and foot screen |  |
|  | Set or modify the SMART Goal and follow up in subsequent visits (refer to [Annex B](#_4i7ojhp) on potential SMART Goals). To check compliance of the resident in self-monitoring diabetes control (eye and foot check, measuring of blood sugar). Ensure that blood sugar measurements are within range. |  |
| Smoking Cessation | | |
|  | For residents who smoke, encourage them to quit smoking using I Quit Programme (<https://www.healthhub.sg/programmes/88/IQuit>) |  |
|  | Set or modify the SMART Goal and follow up in subsequent visits (refer to [Annex B](#_4i7ojhp) on potential SMART Goals) |  |
| Weight loss | | |
|  | Encourage residents with BMI 23 and above to get a weighing machine |  |
|  | Monitor weight every month |  |
|  | To set SMART goals with resident related to diet modifications and exercise which will help with weight loss |  |

###

#

# **Role of Healthcare Volunteers**


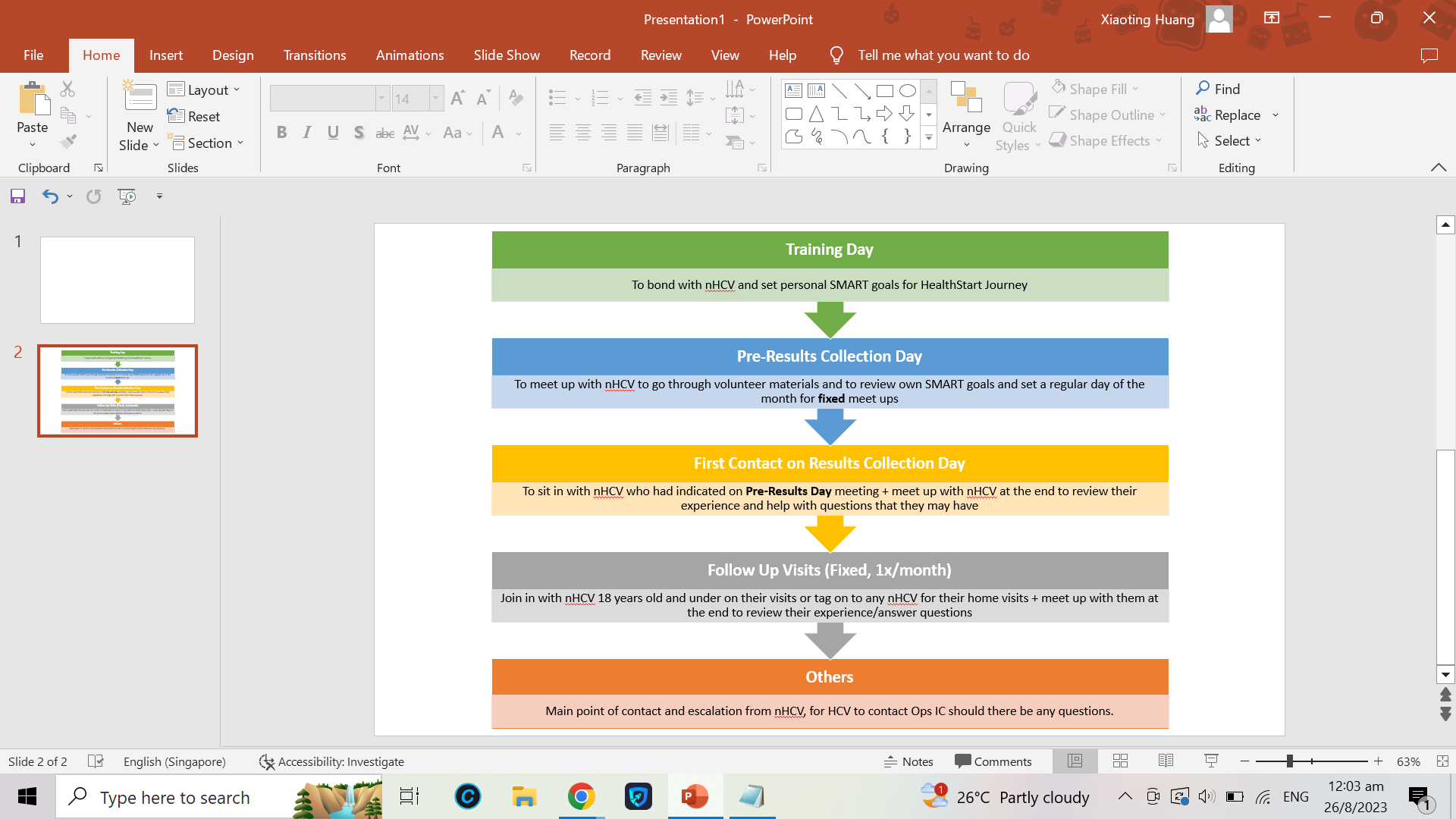


- If the resident did not come for results collection day, explanation of results will be made known to the healthcare volunteer. The healthcare volunteer can proceed to discuss and suggest potential SMART goals to the non healthcare volunteers prior to meeting the resident to disclose the results.
  - Do also note that for residents that did not come for results collection day to collect their results in person, the first visit by the non-healthcare volunteer **must** be accompanied by the healthcare volunteer (in person or over Zoom) to disclose and explain the results.

##

# **Frequently Asked Questions/Potential Scenarios Encountered**

Results Collection Day

1. If residents are not willing for volunteers to join in for community nurse results disclosure

- To explain to residents reason for sitting in is to be better able to understand their health status
- To also minimize duplicity of information shared

Results Disclosure (For residents who did not collect results physically)

1. Unable to contact patient to schedule a visit
   - Kindly follow the diagram below on how to proceed
   - *
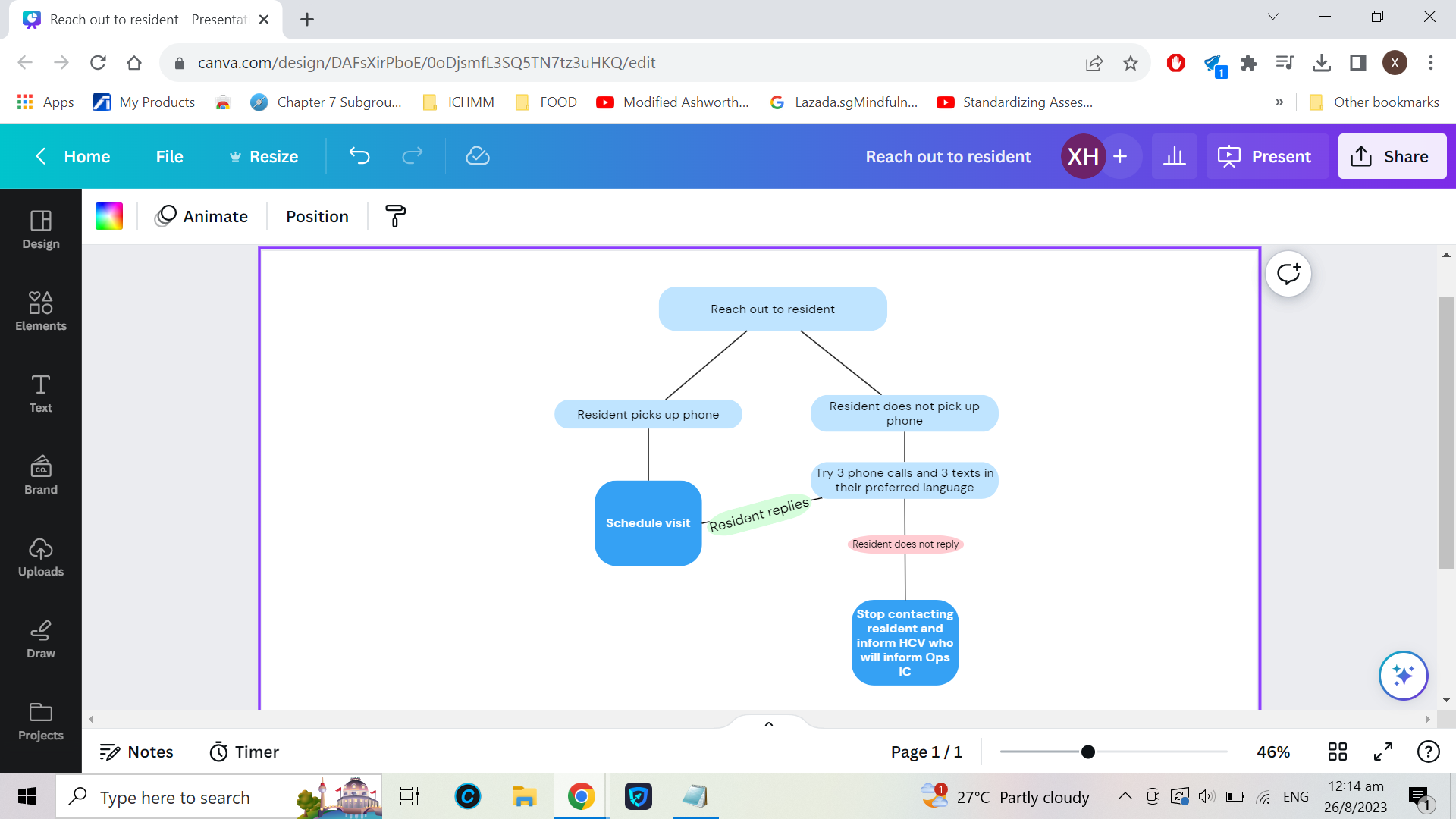
*
2. Resident is overseas
   - Ask if they are agreeable for a follow up when they are back
   - Do schedule a result disclosure date with the resident even if they come back past the mid way of the cycle.
   - Highlight this to your healthcare volunteer.

Follow up Visit

1. Residents changed their minds regarding follow up visit

- Ask if they are agreeable for a follow up call/ to chat via WhatsApp instead of a physical visit
- Explore reasons why residents are not keen for a follow up visit
  - If not keen at all, to discontinue from follow up
  - If keen, to call back again in 1 week to follow up on the resident’s decision
- Inform overall healthcare volunteer of the outcome of the visit

1. Resident was not newly diagnosed with this medical condition. It is an existing medical condition and the resident is taking medications for it. What should I do?
   - If the resident is already diagnosed with the medical condition/taking medications for the medical condition, please check if the resident is currently following up with anyone on this condition.
   - If their health screening results show an abnormal/borderline high value, it means their medical condition is not controlled and can be improved further.
   - If the resident has been lost to follow up, kindly schedule an appointment for the resident to see a doctor (the same one they were seeing previously or polyclinic/GP) to follow up on their condition.
   - If the resident is currently following up with the doctor for that medical condition (abnormal sugar results and following up with a doctor for his/her diabetes), there is no need to schedule a new appointment to see a doctor. However, as their medical condition is not controlled, engage health coaching with the resident to see where they can modify their lifestyle to better control their condition.
   - If the resident is currently following up with a doctor for a different medical condition (abnormal sugar results but following up with a doctor for his/her hypertension), there is no need to schedule a new appointment to see a doctor but advise the resident to bring their health screening results to their doctor the next visit to seek medical advice on the abnormal sugar readings.
2. Resident is overseas
   - Ask if they are agreeable for a follow up when they are back
   - To follow up with the resident even if they come back past the mid way of the cycle. Do highlight to your healthcare volunteer when the resident was overseas/when you begin following up with the resident.
3. Resident does not want to follow up with a GP/ Polyclinic
   - Explore reasons why the resident is not keen for a follow up
   - Clear any misconceptions the resident has and allay their concerns if any
   - If unable to encourage the resident, discuss with the healthcare volunteer in the group possible solutions.
4. My groupmate I am paired with is unresponsive, can I visit my resident alone?
   - As there are group based follow ups, ask if anyone else in the group such as the healthcare volunteer if they will be able to follow you for visits.
   - For adhoc individual follow up, try to schedule WhatsApp calls/contact via WhatsApp to follow up with your resident instead if no one is free to go down with you.
   - We discourage physically visiting the resident alone for non-healthcare volunteers =< 18 years old.

Final Visit

1. I forgot to fill the [final visit form](https://for.sg/hs3finalvisit) during my last visit
   - Please call the resident and ask the questions via phone
   - If the resident is capable of filling the form themselves, kindly send the final visit form to the resident for them to fill up.

# **Escalation of Challenges/Feedback/Ideas**

- For issues regarding content clarification of healthcare issues, to raise to the healthcare volunteer in your group.
- For issues regarding safety/ non-healthcare problems faced, to raise directly on main group chat with TriGen committee members.

# **Annexes**

## **Annex S1: Pre-Training Materials**

The pre-training materials can be found [here](https://docs.google.com/forms/d/e/1FAIpQLSdWCKFMXw4_c653S_ryDmqY8lAFeGXo-DbHm3tE1zyJzm7uBw/viewform?usp=send_form). Please complete it before the in-person volunteer training.

## **Annex S2: Health counseling modules/goal setting**

**Health Goals & Action Plans Guide**

Check the boxes of the goals you would like to work on to manage your chronic conditions in the follow up visit FormSG.

The goals can be summarised with the mnemonic SHEDS My Weight:

1.***S****eeking Help Goals: Visiting Doctor, visiting counsellor*

2.***H****ealth Literacy Goals: Reading more about something, learning new digital apps*

3.***E****xercise Goals: Introducing specific exercise regimes*

4.***D****ietary Goals: Eating/less more of specific groups of food items*

5.***S****moking Goals: Smoking reduction*

*6.****M****onitoring Goals: Watch Blood glucose, watch symptoms*

7.***W****eight Goals: Weight reduction*

My personal goals to reach my target:

| **HEALTH GOALS** | **POSSIBLE ACTION PLANS** |
| --- | --- |
| **Seeking Help Goals (Going to see doctor)** | |
| Ensure regular follow-up with my Doctor & attend annual health screenings | I will book an appointment with my GP/polyclinic to follow up on the conditions I have been diagnosed with |
| **Dietary Goals** | |
| Following a healthy eating plan | To follow a healthy eating plan, I will:   - Drink 6-8 glasses of water a day (unless contraindicated) - If for sugary drinks, I will go for diet or ‘zero’ options - I will go for ‘siew dai’ or ‘kosong’ options when ordering tea/coffee. - I will use My Healthy Plate as a guide for portion estimation (¼ whole grains, ¼ meat and others and ½ with fruits and vegetables)   To eat more wholegrains, I will:   - Change to brown/mixed rice - Change to whole grain bread options   To eat more fruits and vegetables, I will:   - Make sure at least half my plate are vegetables and fruits - Snack on vegetables and fruits, not chips and candy - Have 2 servings of fruits per day - Choose whole fruit over fruit juice - To follow a healthier way of protein intake, I will:   - Choose lean meat, plant protein & dairy products such as egg, fish, chicken and tofu   - To include beans, legumes and dairy (milk, yogurt) |
| Make healthier food choices | To limit alcohol and added sodium, sugar and fat, I will:   - Avoid alcohol or limit to 1 drink/ day (women) & 1 drink/ day (men) - Choose healthier unsaturated fats and oils (oils that have the healthy pyramid label on them) to cook my food   Less sugar & sugary foods:   - Replace sugary snacks with a portion fruit, wholemeal crackers or a glass of milk   Less fat & fatty foods:   - Choose steamed or boiled over fried options - Limit intake of fried dishes to once per week - Avoid butter, ghee or coconut oil in cooking - Choose clear soup as contains less fat   Less salt & salty foods:   - Limit additional table salt at meals, pickled food and bottled sauces - Use less preserved food such as salted fish & canned meats - Use more fresh products   More high fibre foods:   - Switch to unpolished brown rice, whole grain cereals & breads - Include fresh fruit and vegetables |
| **Weight Management Goals** | |
| Monitoring my body weight  (BMI >23kg/ m2 = Overweight) | I will monitor my body weight weekly |
| Taking care of my feet & skin every day | To care for my feet and skin, I will:   - Check feet daily for blisters, hard skin, wounds, sudden skin color changes or cracks in skin - Moisturize feet daily - File any build of hard skin or corns - Cut toenails straight across and smoothen nail edges with a nail file - Wear shoes with supportive features & a good fit |
| **Blood Glucose Monitoring Goals (Monitoring goals)** | |
| [Monitoring my blood glucose with a blood glucose monitoring daily](https://www.healthhub.sg/sites/assets/Assets/Programs/WOD/PDFs/language/en/DiabetesHub-En-04-TakeControl.pdf) | To monitor my blood glucose, I will:   - Check my blood glucose 3 times/ day - Eat/ Drink something sugary if my blood glucose is lower than 4mmol/L |
| Keeping my blood sugar under control | - Aim for blood sugar level between 6-10mmol/L - See a doctor immediately if blood sugar level is >20mmol/L |
| **Watching for Symptoms Goals (Monitoring goals)** | |
| Looking out for symptoms and when to contact my healthcare provider:  Hyperglycaemia  Hypoglycaemia  Diabetes Complications | If I am experiencing Hypoglycemia, I will:   - Know the symptoms when to seek earlier medical attention - Take 15 g of carbohydrates by eating or drinking fast-acting carbohydrates immediately such as:   - 2 sweets   - 3 teaspoons sugar with half cup water   - ½ can soft drink (150-200ml)   - ½ glass fruit juice (150ml) - Avoid these types of foods to treat low blood glucose   - Foods containing fat/ protein, items that requires a lot of prolonged chewing/ sucking - Know the symptoms when to seek earlier medical attention   If I am experiencing Hyperglycemia, I will:   - Take my medications regularly on time and at the same time each day - Aim to have 3 meals daily consistent in timing & portion (Healthy Food Plate) - Cut & minimise snacking - Include regular physical activity as much as I can - Stay well-hydrated and drink water when thirsty. - Avoid sugar-sweetened beverages - Keep a blood glucose and food diary - Know the symptoms when to seek earlier medical attention   To reduce the risk of diabetic complications (Eye – Blindness), I will:   - Keep my blood glucose levels in check - Attend regular eye screening - Treat & keep other medical conditions (High blood pressure, high cholesterol and heart disease under control) - Quit smoking & exercising regularly - Go for annual eye screening - Know the symptoms when to seek earlier medical attention   To reduce the risk of diabetic complications, I will:   - Keep my blood glucose levels in check - Quit smoking & start exercising regularly (at least 150 min/ week). - Keep my blood pressure in the target range with lifestyle measures & medications - Take the medications prescribed by Doctor regularly - Look out for common symptoms such as tingling, burning and pricking sensation or numbness of the hands and feet - Look out for wounds or injuries to my limbs - Go for annual foot screening |
| **Exercise/ Activity Goals** | |
| Exercising & Staying active | To increase activity, I will:   - Walk 30 mins 5 times in my neighbourhood or at a mall - Sign up for an exercise class or fitness programme at a fitness centre or community centre - Achieve 10,000 steps/ day - Do strength training with light weights or without weights - Swim or do a water exercise 30 mins 5 days a week |
| **Other Personal Goals** | |
| Monitoring my **blood pressure**  (Guide: Less than 140/90mmHg or lower in some cases)  (Monitoring Goals) | To monitor my blood pressure, I will:   - Have my blood pressure be measured and recorded regularly - I will find out my blood pressure target |
| Quit smoking (**S**moking Goals) | I will get support and advice on how to quit:   - Asking my Doctor on smoking cessation programs or call 1800-Quit-Now |
| Stress management | I will monitor my own emotional well being and seek help if anytime:   - My appetite is affected - My sleep is affected - My focus is affected |
| Health Literacy Goals | I will read up more about my condition.  I will learn about how to use a health-related digital app to improve my health. |

Exemplar Templates for goal setting

| **Goal Category** | **Example of Template for Goal Setting** |
| --- | --- |
| Seeking Help Goals | 1. I will make an appointment to see a doctor, at _____polyclinic, on ____, for my following health conditions: ____. |
| Health Literacy Goals | 1. Reading: I will read the _____(insert reading material) to find out more about ____ by _____ (Date).    1. Eg. I will read the ScreenForLife Brochure to find out more about my screening requirements by next Monday. 2. Digital: I will use the ____(app) to _____(Insert action, by___(insert date).    1. Eg. I will use HealthHub to arrange an appointment by next Monday. |
| Exercise Goals | 1. I will do ___(specific exercise activity), _____(insert frequency), on _____(insert days of the week), for ___ (duration), starting from ___(date)    1. Eg. I will climb the stairs to my flat, 3x a week and once a day, on Monday, Wednesday and Friday, for 1 month, starting from next Monday. |
| Dietary Goals | 1. I will _____(*Add*/*remove*/*substitute* dietary item), ___(insert frequency), on ____(insert days of the week), for ____(duration), starting from___ (date).    1. Eg. I will drink low fat milk instead of Full cream milk, 5x a week, from Monday to Friday, for 6 months, starting from next Monday. |
| Smoking Goals | 1. I will ___(cut how many sticks), by _____(date), for ___(duration).    1. Eg. I will cut from 15 to 10 sticks by next Monday, for 3 months. |
| Monitoring Goals | 1. I will monitor my ____ (eg. Blood pressure/sugar) ____ (insert no. of times per day) |
| Weight Goals (this **needs to be paired** with either dietary and/or exercise goals. | 1. I am to lose ____(insert weight) by _____(end date). I aim to do this by losing ____(insert weight) every ___(insert time frame). I will do this by (Insert SMART Dietary goal/exercise goal). And I will check in with _____(insert helping professional) by ____(insert date), to see if my plan is working.    1. Eg. I aim to lose 6kg by 6 months. I aim to do this by losing 1kg every month. I will do this by taking the stairs once a day, everyday, for the next 6 months, and avoiding sugar/canned drinks for the next 6 months. And I will check in with my family doctor in 6 month’s time, to see if my plan is working |

##

## **Annex S3: Health-related phone apps**

| **Category** | **Application** |
| --- | --- |
| Medication management | Medisafe Pill Reminder; available on Android and iOS |
| Exercise and nutrition | Healthy365; available on Android and iOS   \| Steps to pair fitness tracker \| <https://www.healthhub.sg/sites/assets/Assets/Programs/NSC-Main-Full/season5/pdfs/H365_Step_by_Step_Guides_How_to_step_up_your_HPB_fitness_tracker_and_other_fitness_tracking_mode.pdf> \| \| --- \| --- \| \| What to do if unable to pair fitness tracker \| <https://www.healthhub.sg/sites/assets/assets/programs/nsc-main-full/season6/pdfs/support/unable-to-pair-your-fitness-tracker.pdf> \|   My Fitness Pal; available on Android and iOS |
| Diabetes management | Diabetes:M; available on Android and iOS |
| Singapore Health Apps | **Singpass App (How-to Guide)**  [https://www.imda.gov.sg/residentsgodigital/-/media/residents-Go-Digital/PDF/pdf3/SingPass%20Mobile_Eng.pdf](https://www.imda.gov.sg/seniorsgodigital/-/media/Seniors-Go-Digital/PDF/pdf3/SingPass%20Mobile_Eng.pdf)  **Healthhub App (How-to Guide)**  [PowerPoint Presentation (imda.gov.sg)](https://www.imda.gov.sg/seniorsgodigital/-/media/Seniors-Go-Digital/PDF/pdf9/HealthHub-Eng.pdf)  **HealthBuddy App (How-to Guide)**  <http://www.singhealth.com.sg/patient-care/patient-visitor-info/health-buddy-app#download>  **Healthy 365 App (How-to Guide)**  [H365 events registration guide.pdf (healthhub.sg)](https://www.healthhub.sg/sites/assets/Assets/PDFs/HPB/H365%20events%20registration%20guide.pdf)  ***Zoom***  Please also download the Zoom app on your phone to facilitate virtual visits with the residents. |

## **Annex S4: Online health resources**

*General Resources and Information*

| **Resource** | **Link** |
| --- | --- |
| Live Well, Age Well Programme | <https://www.healthhub.sg/programmes/160/AAP> |
| Stay Well to Stay Strong | <https://www.healthhub.sg/programmes/170/StayWell> |
| Screen for Life | <https://www.healthhub.sg/programmes/61/Screen_for_Life> |
| I Quit Programme | <https://www.healthhub.sg/programmes/88/IQuit> |
| Colorectal Cancer Screening | <https://www.healthhub.sg/programmes/172/colorectal-cancer-screening> |
| Breast Cancer Screening | <https://www.healthhub.sg/programmes/174/breast-cancer-screening> |
| Diabetes Hub | <https://www.healthhub.sg/programmes/162/diabetes-hub> |
| Council for Third Age | <https://www.c3a.org.sg/> |

*Exercise*

| **Location** | **Resource** | **Link** |
| --- | --- | --- |
| At home | Health Promotion Board Exercise Programmes | <https://youtu.be/4UCkKDlXYk4> (English)  <https://youtu.be/1Z_VH-uGKIM> (Mandarin)  <https://youtu.be/P_UQdfd0jmE> (Malay)  <https://youtu.be/XDWzcpmAxRU> (Tamil) |
|  | Anytime, Anywhere Workout | <https://youtu.be/QA6X7VJklG4> |
| At a Centre | GymTGymTonic Senior Gymonic Senior Gym | <https://gymtonic.sg/> |
|  | NTUC Health Senior Gym | [*https://ntuchealth.sg/active-ageing/services/senior-gym*](https://ntuchealth.sg/active-ageing/services/senior-gym) |

*Nutrition*

| **Resource** | **Link** |
| --- | --- |
| Nutritional Guide | [https://www.healthhub.sg/sites/assets/Assets/Programs/resident-health-nutrition/pdf/Nutrition_Guide-The_Recipe_For_Healthy_Ageing_(English).pdf](https://www.healthhub.sg/sites/assets/Assets/Programs/senior-health-nutrition/pdf/Nutrition_Guide-The_Recipe_For_Healthy_Ageing_(English).pdf) |
| Recipe Book | [https://www.healthhub.sg/sites/assets/Assets/Programs/resident-health-nutrition/pdf/Recipe_Book-The_Recipe_For_Healthy_Ageing_(English).pdf](https://www.healthhub.sg/sites/assets/Assets/Programs/senior-health-nutrition/pdf/Recipe_Book-The_Recipe_For_Healthy_Ageing_(English).pdf) |
| My Healthy Plate | <https://www.healthhub.sg/programmes/55/my-healthy-plate> |
| Healthy Recipes | <https://www.healthhub.sg/programmes/54/recipes> |

## **Annex S5: Safety Protocol**

| **When** | **What** |
| --- | --- |
| Prior to visit | 1. For adhoc/individual visits, inform your healthcare volunteer that you are making your home visit or call at least one day beforehand (provide necessary details on timing, address).    1. For physical visits: If you are =<18 years old and your partner is unable to make it on the visit day, to hold off the visit and reschedule. 2. Only schedule a visit if you have been well in general health. Otherwise, postpone any visits if you are unwell. |
| On day of visit | 1. Dress appropriately for the home visit e.g. do not wear expensive jewellery or dress skimpily.    1. Please wear the TriGen shirt and jeans preferably 2. Inform your healthcare volunteer before and after the visit 3. Ensure the main door/gate is not closed when you are in the resident’s house. (Aim to put yourself between the resident and the door for easy access in event of emergency) 4. Study the environment and do a mini risk assessment of the home. For instance, take note if there are any sharp objects lying exposed on the floor. 5. Do not enter the resident’s house if you hear yelling, screaming, glass breaking etc coming from within. Get away from the house to a safe spot and call the police if you feel that your safety is compromised and inform the TriGen mass chat group 6. Postpone visit if you/the resident    1. Has any of the following acute symptoms: Fever, cough, runny nose, sore throat, loss of taste/smell    2. Has come into contact with a confirmed COVID-19 case. 7. Keep home visits to a maximum of an hour as much as possible; if you feel uncomfortable or encounter challenges during/after the visit, to speak to the healthcare volunteer or bring it up to the main chat group 8. Wear your mask at all times. 9. Please do not post any photos/videos on social media |

Safety tips to bear in mind:

1. Be contactable at all times
2. Keep valuables out of sight
3. Dress professionally and functionally
4. Try to keep a clear path to the door

##

## **Annex S6: SingPass Application**

How to apply for SingPass (Page 22 for pin mailer method)

[**https://www.singpass.gov.sg/home/ui/assets/pdf/Singpass_Registration_Guide.pdf**](https://www.singpass.gov.sg/home/ui/assets/pdf/Singpass_Registration_Guide.pdf)

## **Annex S7: Digital Device and Plans Available**

Equipping residents with a digital device

1. New digital device (with monthly plan)

- IMDA Mobile Access for Seniors

| **Eligibility Criteria** | |
| --- | --- |
| Age | 60 years old and above (based on birth year) |
| Citizenship | Singapore Citizen |
| Current beneficiary of the selected government assistance scheme | - MSF’s ComCare Long Term Assistance (LTA); OR - MSF’s ComCare Short-to-Medium Term Assistance (SMTA); OR - HDB’s Public Rental Scheme |
| Others | Not an existing IMDA Home ACcess beneficiary who has received a smartphone |

| **Details of Mobile Access Plans for eligible seniors** | |
| --- | --- |
| Costs | - 2-years mobile plan, at $5.05 per month - Smartphone, starting at $20.00 (1-time cost) |
| What does the plan come with | - Free SIM card registration - Free caller ID - Unlimited incoming local calls - Continued mobile data connectivity, with no excess data charges (limit to mobile data will depend on the telco/ plan selected by the senior) - Other value-added services |

For more information on the telcos and plans available, please visit <https://www.imda.gov.sg/how-we-can-help/mobile-access-for-seniors>

- DigitalAccess@Home

| **Eligibility** | |
| --- | --- |
| Dwelling Type | HDB flat |
| Income | - Monthly Gross Household Income (GHI)^1^ ≤ $1,900 OR monthly Per Capita Income (PCI)^1^ ≤ $650 - Monthly GHI^1^ ≤ $3,400 OR monthly PCI^1^ ≤ $900, if there is a primary school student or person with disabilities in the household - (For applications received before 31 March 2025)   Monthly GHI ≤ $3,400 OR monthly PCI ≤ $900, if household has MOE school-going child/ children^2^ (with none in a primary school) |
| Citizenship | At least one member in the household is a Singapore citizen |
| Others | Existing beneficiaries of subsidised broadband or subsidised devices (under either the Home Access or NEU PC Plus schemes) will not be offered DigitalAccess@Home’s subsidised broadband or subsidised devices respectively |
| *Income calculated as an average of the last 12 months. GHI (Gross Household Income) refers to all employment income, self-employed income, rental income, overtime pay, allowances, cash awards, commissions and bonuses of all members of the household. PCI (Per Capita Income) refers to the average monthly gross household income divided by the total number of household members.*  *Full-time students aged 25 and younger attending a Government/ Government-Aided School, Junior College, Centralised Institute, Independent School, Specialised Independent School, Specialised School, Institute of Technical Education, Polytechnic or MOE-funded Special Education School*  The following online self-assessment tool on <https://go.gov.sg/digitalaccesschecker> can be used to check on the senior’s eligibility | |

| **Details of DigitalAccess@Home for eligible seniors** | |
| --- | --- |
| What does plan come with | - Subsidised broadband: 500Mbps OR 1Gbps - Subsidised device package:   - Laptop: Includes Microsoft Office, 3-year subscription of anti-virus software, and 3-year warranty OR   - Tablet: includes 3-year subscription of anti-virus software, and 3-year warranty |

For more information on the plan, please visit <https://www.imda.gov.sg/how-we-can-help/digital-access-at-home>

1. Donated digital device (without plan)

Should the resident require a digital device but do not meet the eligibility criteria for the plans listed above, TriGen do have some donated smartphones that can be distributed to them. Do check with the residents and see if they are comfortable with a second hand phone and if they are agreeable to proceed, do reach out to any of the TriGen committee members for. However, do note that TriGen only has donated phones available. Residents will need to get their own prepaid or postpaid card. You may wish to introduce some of the phone plans available in Singapore to the resident.

##

## **Annex S8: Summary of forms to fill and other relevant documents**

Click onto the form/ document that you wish to access to be redirected to the form/ document.

| **Form/ Document** | **Description** |
| --- | --- |
| [First visit form](https://for.sg/hs3firstvisit) | Form to fill during the first touchpoint with residents, to be done together with health coaching |
| [Follow up visit form](https://for.sg/hs3followupvisit) | Form to fill during subsequent touchpoints with residents, to submit for each of the follow up session with residents |
| [Final visit form](https://for.sg/hs3finalvisit) | Form to fill during the last touchpoint with residents |
| [HPB resources for high blood pressure](https://drive.google.com/drive/folders/1defcWofZ1WoRY6eaqy76xBKlYX1YtWUx?usp=sharing) | HPB booklet on managing blood pressure, to share with residents during health coaching |
| [HPB resources for high cholesterol](https://drive.google.com/drive/folders/1tMikwPCkWqHwsalvwPWsbTF85not4Q9u?usp=sharing) | HPB booklet on managing cholesterol, to share with residents during health coaching |
| [HPB resources for diabetes](https://drive.google.com/drive/folders/1r9QgoNgLJ2awDt4OwJhNLqVb1ucqPysv?usp=sharing) | HPB booklet on managing diabetes, to share with residents during health coaching |
| [HPB resources to keep blood sugar level healthy](https://drive.google.com/drive/folders/1prRxL-kN0vqGhCtO2H1x6-pJZ1m-p22f?usp=sharing) | HPB booklet on managing blood sugar, to share with residents during health coaching |
| [Summary video](https://www.canva.com/design/DAFrw0d_Nxg/JDIKM7kWmUu8EpO3V9lGgg/watch?utm_content=DAFrw0d_Nxg&utm_campaign=designshare&utm_medium=link&utm_source=publishsharelink) | Summary video of the volunteer HealthStart journey |
| [My Companion Guide to a Healthier Lifestyle (HealthStart)](https://docs.google.com/document/d/1D_H5E1keqWXfeEt5KAFk1mXyzHQFJPah/edit?usp=drive_link&ouid=101009569086612353102&rtpof=true&sd=true) | Companion guide for residents (to pass hardcopy version to residents, softcopy for volunteers’ reference) |
| Progress card (in [My Companion Guide to a Healthier Lifestyle (HealthStart](https://docs.google.com/document/d/1D_H5E1keqWXfeEt5KAFk1mXyzHQFJPah/edit?usp=drive_link&ouid=101009569086612353102&rtpof=true&sd=true)) | Progress card to track the progress of the residents |

**Appendix S2: Case Based Scenarios used during training of nHCVs**

| **Case Scenario** | **Tasks for nHCVs** | **Learning Objectives** |
| --- | --- | --- |
| **Scenario 1:** Mrs Tan is a retired 67 year old woman with no past medical history.  She attended a community health screening event last month. She lives with her husband, and she does not smoke or drink.  At this year’s health screening she is diagnosed with high blood pressure (where the blood pressure reading is > 140/90) and is encouraged to follow up with the doctors at the polyclinic/ GP.  She has agreed for you to follow-up with her as part of the program. | - Take a relevant history of Mrs Tan’s demographic details and lifestyle. Fill in the “First Visit Form” - Find out Mrs Tan’s understanding about being diagnosed with high blood pressure - Educate Mrs Tan about hypertension using the HPB educational material - Set at least one SMART goal to help Mrs Tan adopt a lifestyle change to improve her blood pressure | - Demonstrate competency in taking a relevant history - Educate participant about hypertension - Demonstrate competency in setting SMART goal(s) |
| **Scenario 2:** Since the last visit Mrs Tan has visited the polyclinic once last month and has been prescribed medication. She was told that she would need to see the doctor again in 3 months time but had to rush off the last clinic visit and forgot to make an appointment. She would like your help to teach her how to use HealthHub to make her follow up appointment. | - Find out how Mrs Tan has been coping with the management of her high blood pressure since the last visit - Check on Mrs Tan’s progress with her lifestyle goals - Teach Mrs Tan how to use HealthHub to manage her chronic condition and make follow up appointments | - Evaluate the participant’s progress in previously set goal(s) - Apply social prescribing skills to enable participants to achieve goals - Demonstrate the ability to teach participants how to use health-related digital applications i.e. HealthHub to help with the management of their chronic conditions |
| **Scenario 3:** Mr Chan is a 62 year old retiree, who is happily married with 3 adult children. He stays with his wife and also fetches his grandchildren home from preschool regularly.  This is your second visit to his home. During your last visit, you shared with him the harms of smoking. He is well aware of it as told by his family and family physician. However he is not keen to change his habit and cites ‘I have been smoking for a long time, it is not easy to change’ and ‘I’ve tried many ways to quit but they are all useless and none of it has worked’ as reasons to continue smoking. On average, he smokes 10 sticks per day. | - Apply Motivational interviewing (MI) principles and skills to motivate Mr Chan to change his smoking habit and elicit a change in behavior - Identify Mr Chan’s current smoking habit - Find out what Mr Chan values as a person - Find out previous efforts to quit | - Be competent in applying principles of motivational interviewing (MI) - Recognize the need for intrinsic motivation in order to effect long term change - Be able to propose strategies for the participant to quit smoking (SMART Goals) |
| **Scenario 4:** Mrs Lim is a 68-year-old living alone in a two-room rental flat and is a recently recovered Covid-19 patient with no past medical history. Recently, she suffered a minor fall at home. Fortunately, she only sustained minor injury and was on follow-up with the polyclinic for her wound treatment. During result collection day, community nurses disclosed to her that she was newly diagnosed with Type II Diabetes Mellitus.  It is your first visit to her house and she appears reserved and quiet when you first enter her flat. | - Find out more about Mrs Lim’s social situation and background - Find out how Mrs Lim is coping and educate her about her diabetes mellitus. | - Demonstrate ability to react appropriately to unexpected situations during home visits (for e.g. participants becoming emotional, aggressive, etc.) - Recognize seniors who may be at risk or have signs of social isolation or depression. - Demonstrate empathy and patience when speaking to a participant with mood issues |

**Appendix S3: Evaluation checklist**

**VOLUNTEER FLAGS**

**FOR CASE BASED SCENARIOS**

**(FOR TRIGEN COMM MEMBERS’ INTERNAL REFERENCE)**

|  | **Competent** | **Will need more training and guidance** | **Not competent** |
| --- | --- | --- | --- |
| **Non-healthcare & Youth Volunteers**  (includes enrolled nurses, medical & nursing students) | - Good communicators, empathetic, enthusiastic - Able to respond confidently to questioning and flow along with conversation - Knows how to pace with residents’ level of acceptance for goal setting and good gauge of residents’ receptiveness to education | - Shy/quiet/passive volunteers - Volunteers that cannot read emotions/not tactful - Dependent on cues given by facilitators during training to complete scenario | - Lack of self awareness/integrity - Giving patients wrong information without clarifying even though they were unsure of the answer to the question - Sticking to the script and ignoring residents’ questions/social cues - Stopping education process when resident says that they are not interested in lifestyle changes instead of exploring more |
