# Supplementary material for: Implementation, Challenges, and Outlook of an Intergenerational, Layperson-led, Health Coaching Program (HealthStart): A Pilot Case Study
Source: JMIR Form Res. 2025 Sep 22;9:e76592. doi: 10.2196/76592 (PMC12453286; doi:10.2196/76592)
Supplement: Multimedia Appendix 2 [file formative-v9-e76592-s002.docx]

**Table S1. Suggested Guide for HealthStart visits.**

| **No.** | **Action** | **Done?** |
| --- | --- | --- |
| Section 0: Prior to Results Collection Day | | |
| 1 | Discuss with your group the dates on which your team plans to do a team follow up and note down the dates. Plan the dates for the follow up 6 visits - total of 3 team visits and 3 adhoc individual visits. |  |
| 2 | Ensure familiarity with disease conditions (hypertension, hyperlipidaemia, diabetes) and the lifestyle modifications/social prescribing that can help with these disease conditions. |  |
| 3 | Ensure familiarity with the [first visit form](https://for.sg/hs3firstvisit) prior to results collection and /or disclosure day. |  |
| 4 | Ensure familiarity in scheduling an appointment on HealthHub / enrolling the resident into Healthier SG |  |
| Section 1a: Results Collection Day (Residents collect in person) (1 Session) | | |
| 1 | Ensure that the resident is aware that they have abnormal health screening results (high blood glucose/high blood pressure/high cholesterol) or have a borderline high result as explained and counseled by the community nurse |  |
| 2 | Complete [first visit form](https://for.sg/hs3firstvisit). The first visit form takes approximately 30 mins to complete and allows you to understand the resident’s eHealth literacy, health ownership, and knowledge of chronic conditions and social history. Set a SMART goal with the resident as well with the first visit form as guidance. |  |
| 3 | Introduce HealthStart to the resident and sign the media consent form. |  |
| 4 | Pass the HealthStart Booklet “My Companion Guide to a Healthier Lifestyle (HealthStart)” to the resident and complete Page 3 of the booklet “Visit 1/Results Collection Day”. Explain to them the “ABC Card” and that this booklet will be used through the HealthStart Journey. |  |
| 5 | Exchange numbers with the resident and inform the resident of the follow up date with the resident. Explain to residents subsequent follow ups can be done with phone calls, [Zoom](https://www.imda.gov.sg/seniorsgodigital/-/media/Seniors-Go-Digital/PDF/pdf5/Zoom_English.pdf)/[WhatsApp video calls](https://faq.whatsapp.com/785056755306362/?locale=en_US)/physical visits. |  |
| 6 | Ask the residents if they have any concerns. Highlight to your healthcare volunteer if the residents are highly distressed about their results. |  |
| Section 1b: Results Disclosure Day (Residents unable to collect in person) (1 Session) | | |
| 1 | Contact the resident and schedule a physical visit with the resident. Ensure the healthcare volunteer in your group is able to attend the visit (physically or virtually). Do also ensure that the resident has received his/ her health screening report prior to the visit. |  |
| 2 | On the scheduled date, the healthcare volunteer is to disclose the health screening results to the residents. Explain how the results can be interpreted (abnormal/ borderline high), complications of the disease condition, and briefly how it can be controlled. |  |
| 3 | After results have been disclosed by the healthcare volunteer, non-healthcare volunteers complete the [first visit form](https://for.sg/hs3firstvisit). The first visit form takes approximately 30 mins to complete and allows you to understand the residents’ eHealth literacy, health ownership, and knowledge of chronic conditions and social history. Set a SMART goal with the residents as well as with the first visit form as guidance. |  |
| 4 | Introduce HealthStart to the resident and sign the media consent form. |  |
| 5 | Pass the HealthStart Booklet “My Companion Guide to a Healthier Lifestyle (HealthStart)” to the resident and complete Page 3 of the booklet “Visit 1/Results Collection Day”. Explain to them the “ABC Card” and that this booklet will be used through the HealthStart Journey. |  |
| 6 | Inform the resident of the follow up date with the resident. Explain to residents subsequent follow ups can be done with phone calls, [Zoom](https://www.imda.gov.sg/seniorsgodigital/-/media/Seniors-Go-Digital/PDF/pdf5/Zoom_English.pdf)/[WhatsApp video calls](https://faq.whatsapp.com/785056755306362/?locale=en_US)/physical visits. |  |
| 7 | Ask the residents if they have any concerns. Highlight to your healthcare volunteer if the residents are highly distressed about their results. |  |
| Section 2: Follow up Visit (5 sessions) | | |
| 1 | For Group follow ups: All members of the group will gather at a meeting point, run through action plan, and raise any concerns before dispersing to visit their residents.    For ad hoc/individual follow ups: Inform your healthcare volunteer the date and time of physical visit or virtual call one day before the visit date. |  |
| 2 | Doctor Follow up visit: Check if the resident has seen the doctor/ set an appointment since diagnosed with abnormal results. Enroll the resident into Healthier SG if not yet enrolled.  ([https://www.healthiersg.gov.sg/enrolment/guide/)](https://www.healthiersg.gov.sg/enrolment/guide/) |  |
| 3 | SMART goal: Based on the disease condition, proceed to the relevant table in the respective section for lifestyle modifications that can be performed to better manage the disease condition. There are guiding questions to ask your resident to aid in setting a smart goal as well as to check their progress with regards to their SMART goal. The relevant sections have useful resources to aid your health coaching.  ● Borderline high or high cholesterol  ● Borderline high or high blood pressure  ● Borderline high or high blood sugar |  |
| 4 | SMART goal: Complete the corresponding visit page (visit 2-6) in HealthStart Booklet “My Companion Guide to a Healthier Lifestyle (HealthStart)”. Set a new SMART goal with the resident if the previous SMART goal has been achieved (refer to Annex B on potential SMART Goals). |  |
| 5 | Digital: Check if residents are keen to learn digital apps (apps include Singpass, HealthHub App, Healthy365)  Refer to Annex G if the resident does not have a digital device and refer to Annex F for how to use the pin mailer to apply for Singpass.  Teach the residents how to track appointments and results, check for health screening eligibility, and make appointments via HealthHub. Do also share with residents on how they can sign up for active lifestyle events and participate in Eat, Drink, Shop program to earn rewards on Healthy365 |  |
| 6 | Optional: advise and encourage your residents to go for further health screening, i.e., age-appropriate cancer screening, adult vaccinations etc. |  |
| 7 | If resident is keen for further follow ups during the visit that you have with him/ her: fill up [follow visit form](https://for.sg/hs3followupvisit) after each follow up visit.  If resident is not keen for further follow ups during the visit that you have with him/ her: fill up [final visit form](https://for.sg/hs3finalvisit). |  |
| 8 | Track the progress of the residents on ABC card and encourage the resident to collect all stamps to receive a goodie bag. Remind the resident of the next visit date. |  |
| 9 | For group follow-ups: Gather at the end of the visits for a meal/drink. Debrief with the healthcare volunteer as a group and ask for advice from each other and the healthcare volunteer on how to better encourage the resident further to meet the HealthStart Goals.  For ad hoc/individual follow up: Message your healthcare volunteer or even your group a summary of the visit and raise any questions/concerns to your healthcare volunteer/group members if needed. Ask for advice from each other and/or the healthcare volunteer on how to better encourage the residents further to meet the HealthStart Goals. |  |
| Section 3: Final Visit and HealthStart Goals to Complete (1 session) | | |
| 1 | Fill up the final visit form. |  |
| 2 | Complete the final visit page (visit 7) in HealthStart Booklet “My Companion Guide to a Healthier Lifestyle (HealthStart)”. |  |
| 3 | Ensure all the following HealthStart goals are completed: |  |
|  | 1. Ensure Health Promotion Board (HPB) booklet(s) on the disease condition(s) have been completed |  |
|  | 1. Achieved at least 1 SMART goal set |  |
|  | 1. Taught at least one digital health app e.g., HealthHub, Healthy365, Healthbuddy |  |
|  | 1. Resident followed up with a polyclinic/GP on his/her newly diagnosed condition and/or enrolled into Healthier SG. |  |
|  | 1. Ensure all follow up visit and final visit FormSG forms are filled |  |
|  | Bonus: advised and encouraged residents to go for further health screening i.e. age-appropriate cancer screening, adult vaccinations etc. |  |

**Table S2. Content of volunteer training.**

| **Youth Community Health Volunteers** | **HealthCare Volunteers (HCV)** |
| --- | --- |
| Pre-recorded lectures: 1) Singapore Healthcare System, 2) Chronic Diseases and Principles of Management, 3) Social Determinants of Health, 4) Vital Signs Monitoring | |
| Icebreakers and Team Building | |
| Briefings: Overview of HealthStart, Operational Aspects of HealthStart | |
| SMART goal setting and principles of motivational interviewing | |
| Revision of chronic disease knowledge using an audience response system | Mentoring and facilitation workshop |
| Case-based scenarios (Four scenarios) | |
